# Microwave-Assisted Enantioselective Synthesis of (2*R*,5*S*)-Theaspirane: A Green Chemistry Approach

**DOI:** 10.3390/molecules30071519

**Published:** 2025-03-29

**Authors:** Sayuri Cristina Santos Takada, Maria Carolina Blassioli-Moraes, Miguel Borges, Raul Alberto Laumann, Izabella Vitória Maravalho, Wender Alves Silva

**Affiliations:** 1 Embrapa Genetic Resources and Biotechnology, Empresa Brasileira de Pesquisa Agropecuaria, Brasilia 70770-917, Brazil; carolina.blassioli@embrapa.br (M.C.B.-M.); miguel.borges@embrapa.br (M.B.); raul.laumann@embrapa.br (R.A.L.); 2 Laboratory for Bioactive Compound Synthesis, University of Brasilia (IQ-UnB), Campus Universitario Darcy Ribeiro, Brasilia 70904-970, Brazil; izabellavitoria22@hotmail.com

**Keywords:** microwave-assisted reactions, (2*R*,5*S*)-theaspirane, semiochemicals, kinetic resolution, green chemistry, cyclization, stereoselective synthesis

## Abstract

The banana weevil (*Cosmopolites sordidus*) is a significant pest that reduces banana yields and can result in plant mortality. (2*R*,5*S*)-theaspirane, a kairomone from senesced banana leaves, is one of the natural banana volatiles, aiding weevil attraction. A rapid and cost-effective synthesis of (2*R*,5*S*)-theaspirane was developed utilizing microwave-assisted conditions and the principles of green chemistry. The process comprised five steps, beginning with the reduction of dihydro-β-ionone, followed by lipase-mediated kinetic resolution to attain high enantiomeric excess. Microwave-assisted heating significantly reduced reaction times. Optimized cyclization with the minimum quantities of selenium dioxide oxidation was employed. The final diastereomers were separated by chromatography, yielding compounds which exceeded 99% enantiomeric purity.

## 1. Introduction

**Figure 1:**
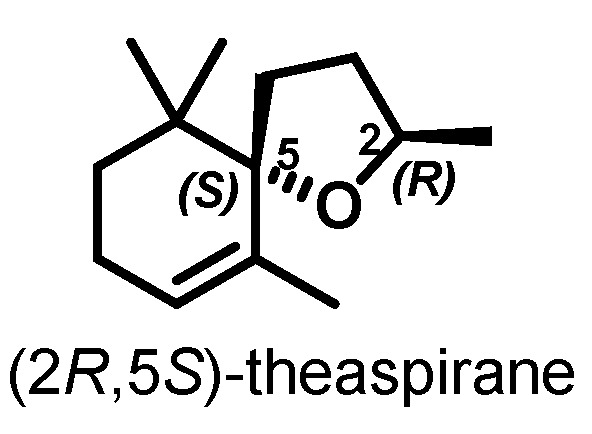
Structure of the kairomone identified from banana leaves that attracts both male and female banana weevils.

Chemical signaling plays a fundamental role in interspecific interactions, with kairomones acting as essential mediators that exclusively benefit the receiving organism, aiding predators in locating prey and parasites in identifying hosts [1]. These compounds are crucial for ecological processes such as predation, parasitism, and plant defense, while also offering potential applications in agriculture and pest management [2]. Among biologically active oxygenated heterocycles, apocarotenoids—such as ionone, damascone, and theaspirane isomers—naturally occur in various fruits and plants [3]. Notably, (2*R*,5S)-theaspirane has been identified as the major volatile compound responsible for attracting the banana weevil (*Cosmopolites sordidus*), a devastating pest in banana cultivation [4] (Figure 1).

Despite the biological and ecological significance of theaspirane, existing synthetic methodologies present several limitations, including low yields, the use of toxic reagents and solvents, expensive catalysts, challenges in isomer isolation, and, in most cases, a lack of stereoselectivity. Consequently, the development of more efficient and environmentally sustainable approaches for its synthesis has become a priority. Recent trends in green chemistry emphasize the integration of safer solvents (e.g., water, ethanol, and supercritical CO_2_), renewable feedstocks, and catalytic processes to minimize waste generation and environmental impact [5]. The combination of microwave technology with these principles holds promise for more sustainable chemical processes; however, critical evaluation is required to ensure that the energy savings and environmental benefits outweigh the equipment and operational costs, particularly in comparison to other emerging green technologies such as flow chemistry [6]. Thus, while microwave-assisted synthesis remains a valuable tool in green chemistry, its widespread adoption must be critically assessed within the broader context of sustainable process development [7].

Microwave-assisted organic synthesis (MAOS) has emerged as a powerful tool in modern organic chemistry, offering significant advantages in terms of reaction rate acceleration, energy efficiency, and improved yields compared to conventional heating methods [8]. Despite these benefits, the application of microwave technology in large-scale processes remains challenging due to limitations in homogeneous heating and reactor design for industrial-scale systems [9]. Furthermore, although microwave irradiation can enhance reaction selectivity and reduce reaction times, its overall contribution to sustainability depends on factors such as energy savings, solvent selection, and process safety [10].

**Scheme 1:**
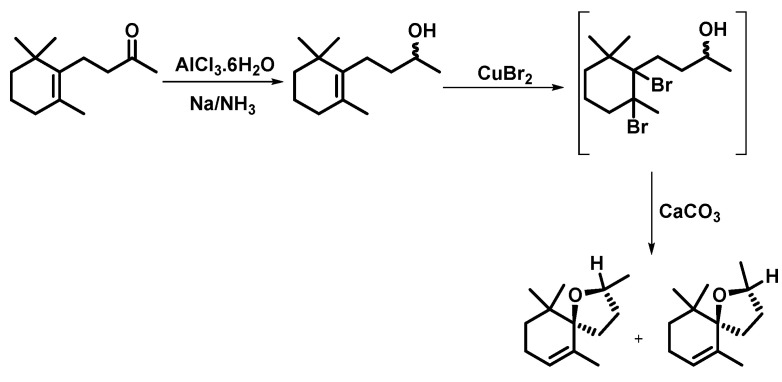
Synthesis of theaspirane involving the use of metal-based compounds.

Masuda et al. (1985) pioneered the synthesis of theaspirane [11]. Although the proposed method is considered efficient, it utilizes hazardous reagents, including aluminum chloride hexahydrate, a sodium–ammonia system, and cupric bromide. Consequently, a large amount of metallic waste is generated (Scheme 1) [12].

**Scheme 2:**
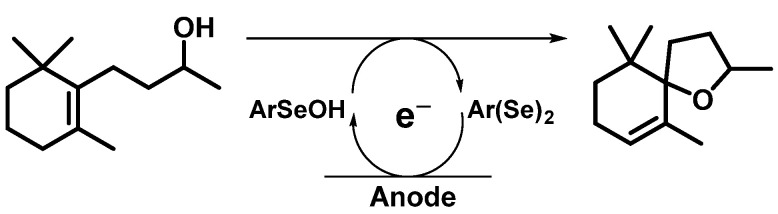
Electrochemistry as a non-stereoselective synthetic pathway for theaspirane.

In the same year, Torii et al. synthesized theaspirane from dihydroionol using electrochemical methods [13]. While the process involves a single step, its associated costs are relatively high due to the use of platinum electrodes and the specificity of the system as a whole. Moreover, other synthetic aspects raise sustainability concerns, particularly the use of toxic reagents such as bis(*p*-chlorophenyl) diselenide, which are frequently used in organic synthesis; however, their toxicity raises significant environmental and health concerns. Like tetraethylammonium perchlorate, they are known for their oxidative properties, but they present substantial safety risks due to their explosive nature and toxicity, including their potential to irritate the respiratory system (Scheme 2) [14,15]. Additionally, the synthetic route lacks selectivity in the formation of stereoisomers.

**Scheme 3:**
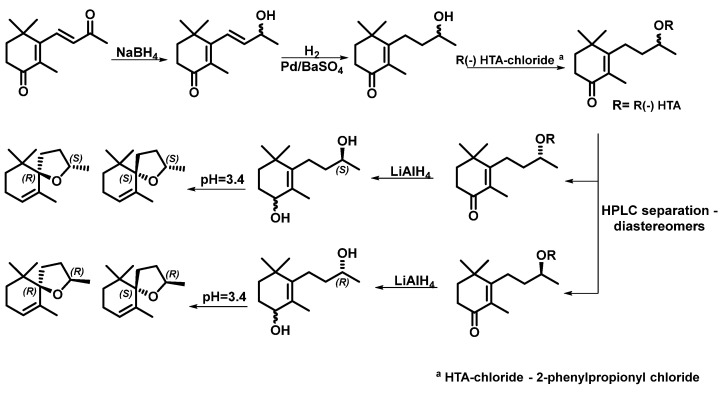
Non-convergent synthesis and enantiodifferentiation of theaspirane isomers.

The first enantioselective synthesis of theaspirane, described by Schreier et al., enabled enantiodifferentiation but employed a non-convergent methodology with low overall yield and limited sustainability [16]. The process relies on multiple reducing agents, such as H_2_/Pd and LiAlH_4_, which are not only hazardous but also increase the risk of accidents. Additionally, the thermal cyclization step occurs under acidic conditions, and the overall synthesis is highly energy-intensive, involving multiple stages that require distillation/extraction, and concentration using a Vigreux column (Scheme 3).

**Scheme 4:**
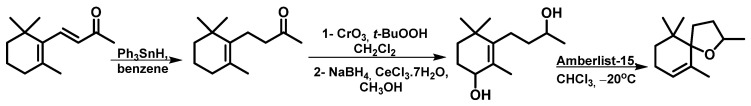
Use of toxic reagents in the synthesis of a banana weevil attractant.

After a period during which methodological studies and synthetic efforts were primarily dedicated to the exploration of theaspirane derivatives, two publications in 2000 introduced new synthetic approaches. The first of these studies, Young et al., synthesized theaspirane in three steps starting from β-ionone [17]. Even though it is a synthetic route and relatively short, it lacks any step that produces theaspirane in a stereoselective manner, as shown in Scheme 4. Furthermore, the reagents used are metal-derived, such as cerium and chromium, as well as peroxides, all of which exhibit a high level of toxicity and pose significant risks of accidents [18].

**Scheme 5:**
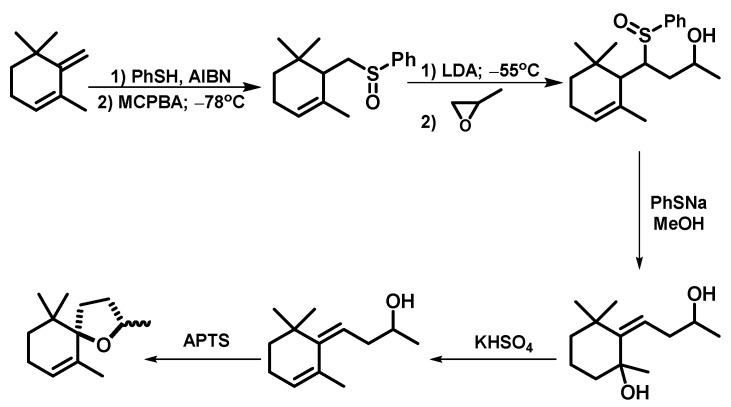
A synthetic route involving potentially explosive materials, posing a significant risk of accidents.

Boullin et al. synthesized a mixture of theaspirane isomers using highly sensitive reagents [19], cryogenic temperatures, and toxic compounds, including azobisisobutyronitrile (AIBN), thiophenol, *m*-chloroperbenzoic acid (MCPBA), diisopropyl lithium amide (LDA), and propylene oxide (Scheme 5). MCPBA is particularly noteworthy due to its explosive potential, making it unsuitable for scale-up reactions without prior thermochemical evaluation [20]. Additionally, their methodology required prolonged reaction times under reflux using a Dean–Stark apparatus.

**Scheme 6:**
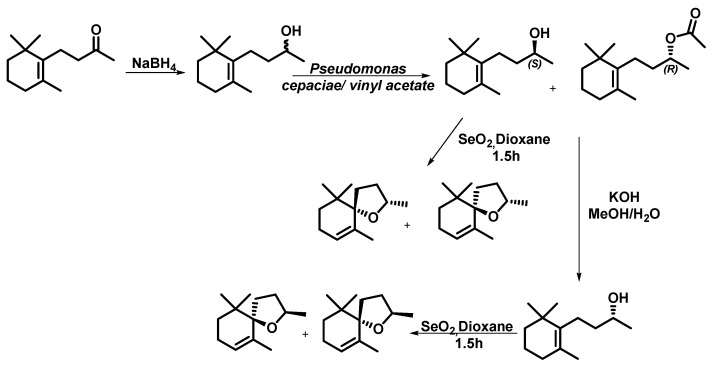
Stereoselective synthesis of theaspirane.

Finally, Braimah et al. unequivocally confirm the stereoisomer that acts as a kairomone for banana weevils in their study [21]. The reported synthesis is stereoselective, achieving high stereoselectivity but with a low overall yield (Scheme 6). However, the authors do not provide a detailed experimental section or analytical data, including chromatograms of the stereoisomers, and they report difficulties in separating the diastereoisomers, which hinders a more critical evaluation of the synthesis of (2*R*,5*S*)-theaspirane.

Herein, we present an environmentally friendly and innovative synthetic approach that integrates microwave-assisted reactions with green chemistry principles, offering an efficient and more sustainable alternative to conventional methodologies. By replacing traditional reagents and solvents, our strategy enhances yields and stereoselectivity while significantly reducing reaction times and environmental impact. Moreover, this approach facilitates the scalable and sustainable production of (2*R*,5*S*)-theaspirane, a compound of agricultural significance with promising applications in pest control.

## 2. Results and Discussion

### Microwave-Assisted Stereoselective Synthesis of (2R,5S)-Theaspirane

**Scheme 7:**
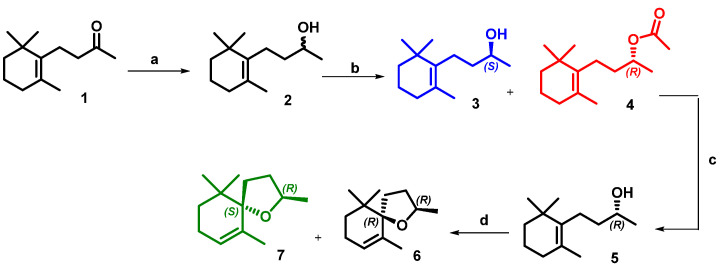
Synthesis of (2*R*,5*S*)-theaspirane. The reagents and reaction conditions: (a) NaBH_4_/EtOH, 60 °C, 10 min, M.W; (b) Lipase Amano PS immobilized on diatomite, cyclohexane 80 °C, 120 min, M.W; (c) NaOH 25%, EtOH, 80 °C, 30 min, M.W; (d) SeO_2_ (2Eq), 2-MeTHF, 80 °C, 30 min, M.W.

As previously described, theaspirane syntheses were not conventionally designed using sustainability concepts. In this work, we carried out stereoselective synthesis for the first time and applied green chemistry concepts with the reactions being assisted exclusively by microwaves (Scheme 7).

**Scheme 8:**
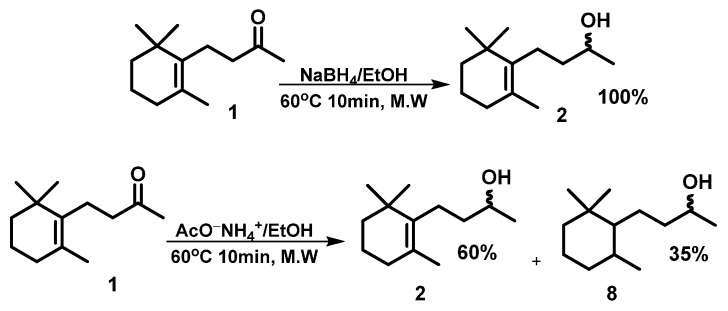
Methodologies for the reduction of β-dihydroionone.

The initial step consists of the reduction of β-dihydroionone (**1**). Although this reaction generally achieves high yields through classical synthesis, it typically requires reaction times on the scale of hours. In this paper, the duration was significantly shortened through microwave-assisted heating. We investigated the influence of different reducing agents by employing ammonium formate, which aligns with the principles of green chemistry, and sodium borohydride, a conventional reagent usually used for this purpose. The ammonium formate methodology tested [22] lacked selectivity, as it also reduced other unsaturated bonds in β-dihydroionone, resulting in a mixture of products with moderate efficiency. When sodium borohydride was used, it furnished non-stereospecific dihydro-β-ionol with a quantitative yield and sufficient purity for direct use without requiring additional purification, as shown in Scheme 8.

**Scheme 9:**
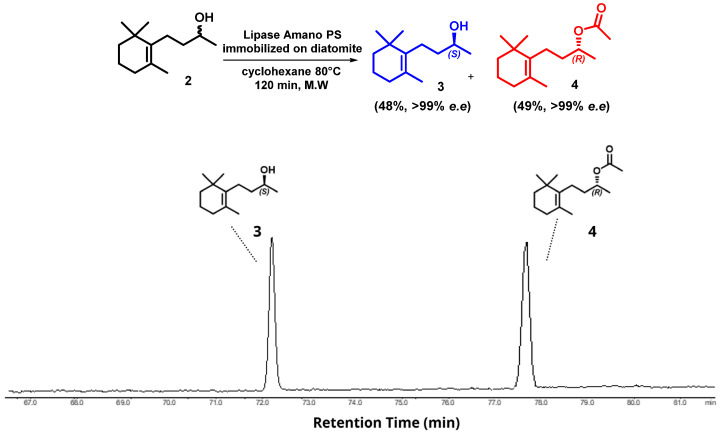
Chemoenzymatic resolution of racemic dihydro-β-ionol yielding (*S*)-dihydro-β-ionol and (*R*)-dihydro-β-ionol acetate, with chiral analysis by GC-FID.

Next, we directed our attention to the chemoenzymatic resolution of alcohol (**2**), as illustrated in Scheme 9. The mixture of dihydro-β-ionol isomers was successfully resolved utilizing Amano Lipase PS immobilized on diatomite, with vinyl acetate serving as the acyl donor in cyclohexane. This process yielded (*S*)-dihydro-β-ionol with a 48% yield and >99% enantiomeric excess (e.e), as well as (*R*)-dihydro-β-ionol acetate with a 49% yield and >99% e.e. For further details, refer to the Supporting Information. The reaction was conducted under heating conditions without any loss of enzymatic activity. Compared to conventional methods, which typically require mild heating (~30 °C) and prolonged reaction times—sometimes extending several days under orbital shaking, depending on the substrate—this approach significantly reduced the reaction time and enhanced enantiomeric excess. Additionally, this methodology employed a more environmentally friendly solvent, minimizing waste generation, as the reactions were carried out in toluene or hexane. With these modification, excellent yields and enantiomeric excesses were achieved. The lipase B from *Candida antarctica* (Aldrich-Merck, St. Louis, MO, USA) was also evaluated; however, it exhibited lower enantioselectivity (yield 45%, 86% e.e, (*S*)-dihydro-β-ionol and yield 43% 94% e.e, (*R*)-dihydro-β-ionol acetate), GC analysis using a chiral column (Beta DEX™ 110, 30 m × 0.25 mm, Supelco, St. Louis, MO, USA).

**Scheme 10:**
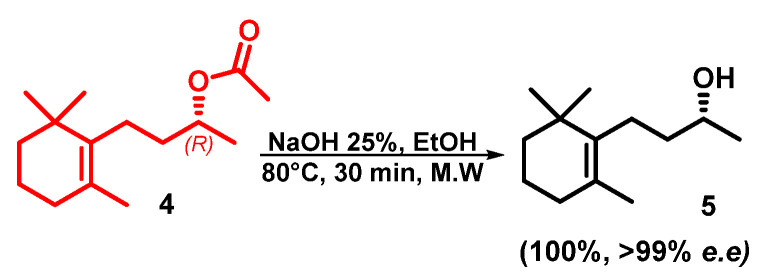
Hydrolyses of (*R*)-dihydro-β-ionol acetate under microwave-assisted kinetic resolution.

For the synthesis of the (2*R*,5*S*)-theaspirane isomer, only the (*R*)-4 compound was used. This compound was hydrolyzed with 25% NaOH in ethanol at 80 °C, yielding alcohol (*R*)-5 in a quantitative yield with a high enantiomeric excess and enantiomeric ratio (E) (100%, >99% e.e, E = 15,198); the enantiomeric ratio was calculated according to Gawley [23]. The microwave-assisted kinetic resolution demonstrated significantly higher selectivity than the method reported by Braimah et al. (E = 1377) [21]. In contrast, our methodology achieved E = 15,198, indicating a superior optimization of the solvent/temperature/enzyme/substrate system. Additionally, this method also provides a significant advantage over conventional hydrolysis by reducing reaction time [24]. While conventional hydrolysis typically requires several hours, this approach shortens the reaction time to just 30 min (Scheme 10).

The final step was the most challenging. Reports on cyclization using SeO_2_ often do not provide a precise description of the methodology employed, offering only a general indication of how the process was conducted [25,26,27,28]. Consequently, a thorough study was necessary to determine not only the optimal experimental conditions but also to minimize the use of SeO_2_ quantities and reduce the waste generation; the results are described in Table 1.

Initially, we carried out the cyclization reaction under the same conditions (time and solvent) reported by Braimah et al. [21]. Since no information was provided regarding the stoichiometric ratio of SeO_2_, two conditions were tested: one with 1 equivalent and the other with 2 equivalents under conventional heating (entries 1 and 2, respectively). Notably, the yields were extremely low. Based on these results, we modified the heating method by employing microwave-assisted conditions to optimize the relationship between reaction time and stoichiometric ratio, thereby improving the yield. Minimizing SeO_2_ use is essential to reducing waste. Another critical modification involved the choice of solvent. We replaced the commonly used dioxane with 2-methyltetrahydrofuran (2-MeTHF), a more sustainable and environmentally friendly alternative derived from renewable biomass sources. Additionally, 2-MeTHF is more hydrophobic than dioxane, particularly during extraction and purification.

The temperature and the amount of SeO_2_ are key factors contributing to the low yield previously described. Therefore, we decided to investigate the reaction at shorter times (15 and 30 min) while using relatively small stoichiometric quantities. With this approach, we initiated our study under conditions similar to conventional heating. By comparing the results of entries 1 and 3, we observed that although the yield was not yet satisfactory, it already showed an improvement compared to the reaction conducted under conventional heating conditions. When the reaction time was increased while maintaining one equivalent of SeO_2_ (entry 4), the yield did not vary significantly.

Through variations in temperature, reaction time, and SeO_2_ stoichiometry, the best result was obtained in entry 10, achieving a yield of 73%. Under optimized conditions, the cyclization reaction demonstrated high efficiency, emphasizing the critical role of controlled temperature and precise molar quantities of SeO_2_. However, in entries 11–16, where both the temperature and the amount of SeO_2_ were increased, the desired product was not obtained, and significantly lower yields were observed. Thin-layer chromatography (TLC) monitoring revealed the formation of an additional product in these entries, which was likely a decomposition compound. Despite isolation attempts via column chromatography, this product could not be identified by ^1^H NMR analysis. These results underscore the sensitivity of the reaction to excessive temperature and reagent quantities, which appear to promote decomposition rather than the intended cyclization. In contrast, entries 1–8, which employed a significant excess of reagents, did not exhibit this issue, further highlighting the importance of maintaining optimal reaction conditions.

To evaluate whether the established conditions were applicable to gram-scale synthesis (scale-up), an experiment was conducted using racemic alcohol (**2**) to synthesize racemic theaspirane in a larger microwave reaction vessel (20 mL capacity), as described in the Materials and Methods section. The product was obtained in 70% yield, demonstrating that no significant variation was observed compared to the reaction performed on the milligram scale.

**Scheme 11:**
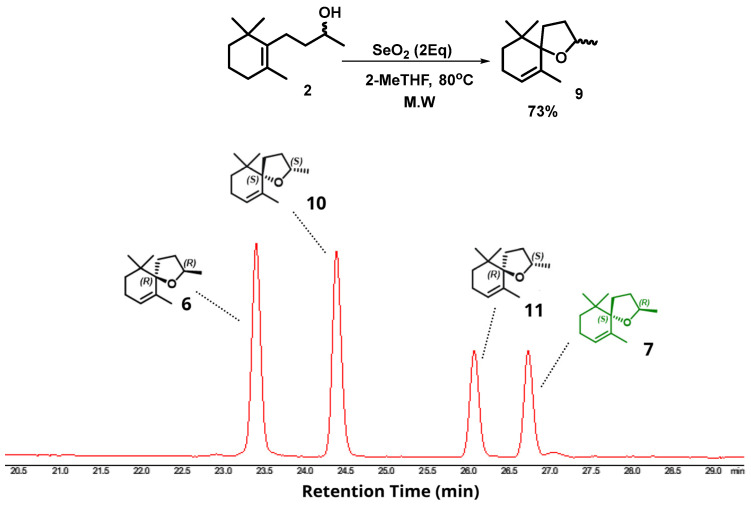
Intramolecular cyclization and chiral analysis (GC-FID) of the four synthesized theaspirane isomers.

Once the optimized reaction conditions were established, we proceeded with the synthesis, exploring the chiral aspects by analyzing the enantiomeric and diastereomeric excesses of theaspirane isomers. GC analyses were performed using a chiral column (Beta DEX™ 110, 30 m × 0.25 mm, Supelco), with the identification of the four stereoisomers conducted in accordance with the work of Braimah (Scheme 11) [21].

**Scheme 12:**
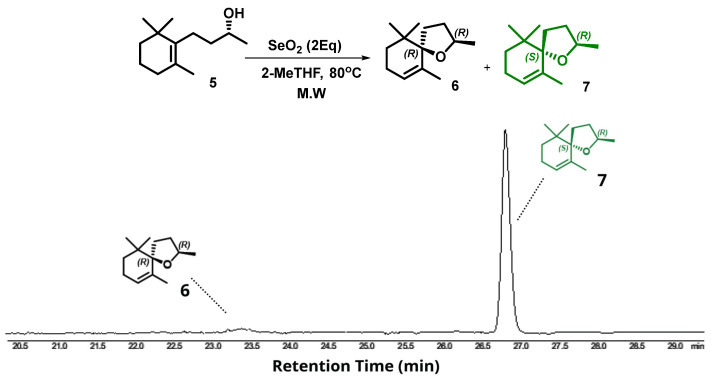
Intramolecular cyclization and chiral column (GC-FID) analysis to generate diastereomeric theaspirane isomers.

After the hydrolysis described in Scheme 10, alcohol (*R*)-5 was subjected to cyclization conditions with SeO_2_, yielding a diastereomeric mixture of (2*R*,5*R*) and (2*S*,5*R*) with a 71% yield and >99% diastereomeric excess (d.e) and e.e. The GC-FID analyses were performed using a chiral column (Beta DEX™ 110, 30 m × 0.25 mm, Supelco), as shown in Scheme 12.

The separation of diastereoisomers was a critical step, with the main challenge arising from the low polarity of the compounds. The crude product was purified by column chromatography on neutral alumina using a gradient elution of hexane and ethyl acetate, ranging from 100:0 to 90:10.

**Figure 2:**
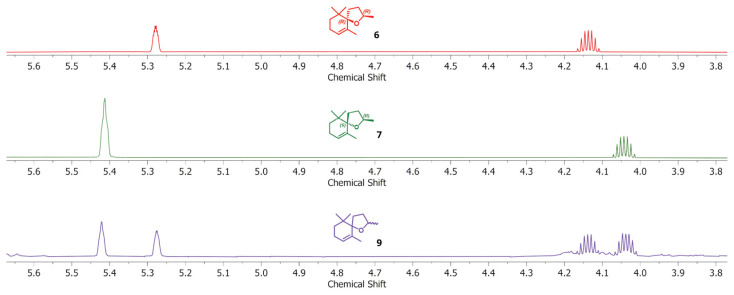
Comparison of the chemical shifts in the diagnostic regions (chiral centers) in the ^1^H NMR spectrum. All compounds were dissolved in CDCl_3_, with TMS used as the reference.

Following the isolation step, ^1^H NMR spectra were obtained to compare the characteristic signals of the isomer (2*R*,5*R*)-theaspirane (**6**), the isomer (2*R*,5*S*)-theaspirane (**7**) and the mixture of diastereoisomers of the theaspirane (**9**), as shown in Figure 2. The significant differences in chemical shifts in the regions of the chiral centers clearly indicate not only the purity of the product but also unequivocally confirm the identity of the obtained isomer.

Finally, through a comparison of our methodology with previously reported approaches outlined in this study, we highlight significant advancements in the field of green chemistry. Our strategy demonstrates significant improvements, including the rationalization of toxic reagent usage, the selection of safer solvents, enhanced atom economy, the design of safer chemicals, improved energy efficiency, and a strong emphasis on accident prevention and waste minimization. These contributions represent meaningful steps toward more sustainable and environmentally responsible synthetic methodologies.

The comparative analysis considers reaction conditions, yields, enantiomeric excess values, and, when applicable, enantiomeric ratio (E), as presented in Table 2. These parameters provide a comprehensive evaluation of the efficiency and environmental impact of our methodology (entry 6, microwave-assisted conditions only), relative to conventional synthetic routes.

## 3. Materials and Methods

All reagents and solvents were purchased from Sigma-Aldrich Merck (St. Louis, MO, USA). The microwave reactions were performed on a Biotage Initiator+ (Uppsala, Sweden), microwave reactor using sealed vessels, a dynamic program, temperature detection by internal fiber optic probe, simultaneous cooling, and media stirring. The NMR spectra were recorded at 25 °C on a Bruker Avance 600 spectrometer (Billerica, MA, USA, 600 MHz for ^1^H and 151 MHz for ^13^C) with TMS as an internal standard for deuterated chloroform (CDCl_3_) as solvent. Optical rotation was measured with a Perkin Elmer Lambda 950 UV-Vis-NIR (Waltham, MA, USA). The GC/MS were recorded in a Shimadzu GC-2010 chromatograph (Kyoto, Japan), which was equipped with a 5%-phenyl-95–methylsiloxane (HP-5) capillary column (30 mm × 0.32 mm × 0.25 μm) and utilized helium as the carrier gas at a flow rate of 1.0 mL/min. The oven temperature was programmed to increase from 100 °C to 200 °C at a heating rate of 3 °C/min. Data processing was performed using the GC-MS solution software version 4.45 SP1. For analysis, GC/FID were recorded with a Shimadzu GC-2010 PRO equipped with a Supelco β-DEX™ 110 column (30 m × 0.25 mm × 0.25 μm). The injector temperature was set at 200 °C, operating in Split mode (30:1), with the manual injection of a 1 μL sample. The carrier gas was helium (He) at a constant flow rate of 0.69 mL/min. The detector temperature was maintained at 250 °C. The gas flow rates were as follows: H_2_ = 40.0 mL/min, synthetic air = 400.0 mL/min, and N_2_ = 30.0 mL/min. The melting points were measured with a capillary on a LOGEN Scientific (LS III Plus, Waltham, MA, USA) apparatus. The reactions were monitored by thin-layer chromatography (TLC) on precoated 0.25 mm thick plates of Kieselgel 60 F254 (Burlington, MA, USA); visualization was accomplished by UV light (254 nm) or by spraying a solution of 5% (*w*/*v*) vanillin in 100 mL 20% (*w*/*v*) aq. sulfuric acid and heating at 200 °C for a sufficient duration until blue spots become visible.

### 3.1. Microwave-Assisted Synthesis of Dihydro-β-ionol

A sealed 10 mL glass tube containing a mixture of dihydro-β-ionone (1.0 mmol, 0.21 mL) and sodium borohydride (2.0 mmol, 75.66 mg) in ethanol (5.0 mL) was placed in a microwave reactor (Biotage Initiator+, Uppsala, Sweden) and heated at 60 °C for 10 min under magnetic stirring, the reactions were monitored by thin-layer chromatography (TLC). The reaction mixture was extracted with water and dichloromethane (3 × 10 mL). The combined organic layers were dried over anhydrous sodium sulfate, and the solvent as removed by evaporation under reduced pressure. The product was obtained in a sufficiently pure form and used directly without further purification. (196.2 mg, yield 100%, m.p 38–40 °C). ^1^H NMR (600 MHz, CDCl3) δ (ppm): 1.00 (6H, s), 1.22 (3H, d, *J* = 6.5 Hz), 1.42–1.44 (2H, m), 1.49–1.61 (8H, m), 1.90 (2H, t, *J* = 6.0 Hz), 1.92–1.98 (1H, td, *J* = 2.8 Hz), 2.11–2.16 (1H, td, *J* = 2.8 Hz), 3.78–3.83 (1H, sextet, *J* = 2.1Hz); ^13^C NMR (151 MHz, CDCl_3_) δ (ppm): 19.5, 19.7, 23.2, 24.7, 28.5, 32.7, 34.9, 39.8, 39.9, 68.8, 126.9, 136.8.

### 3.2. Kinetic Resolution of Dihydro-β-ionol by Lipase from Amano Lipase PS Under Microwave-Assisted Conditions

In a sealed 10 mL glass tube, cyclohexane (5.0 mL), vinyl acetate (1.74 mmol, 0.160 mL), Amano Lipase PS (30 mg, 500 U/g, immobilized on diatomite), and dihydro-β-ionol (0.154 mmol, 30 mg) were added. The reaction was performed in a microwave reactor (Biotage Initiator+) and heated to 80 °C for 120 min under magnetic stirring. Upon completion, the lipase was removed by filtration. The filtrate was then concentrated under reduced pressure and purified via column chromatography on silica gel using a hexane/ethyl acetate (9:1) mixture as the eluent, affording enantiomerically pure (*S*)-dihydro-β-ionol (48% yield, >99% e.e, 14.3 mg) and (*R*)-dihydro-β-ionol acetate (49% yield, >99% e.e, 17.9 mg). Both products were obtained as colorless oils.

(*S*)-dihydro-β-ionol ^1^H NMR (600 MHz, CDCl_3_) δ (ppm): 1.00 (6H, s), 1.22 (3H, d, *J* = 6.5 Hz), 1.42–1.44 (2H, m), 1.49–1.61 (8H, m), 1.90 (2H, t, *J* = 6.0 Hz), 1.92–1.98 (1H, td, *J* = 2.8 Hz), 2.11–2.16 (1H, td, *J* = 2.8 Hz), 3.78–3.83 (1H, sextet, *J* = 2.1Hz); ^13^C NMR (151 MHz, CDCl_3_) δ (ppm): 19.5, 19.7, 23.2, 24.7, 28.5, 32.7, 34.9, 39.8, 39.9, 68.8, 126.9, 136.8.

(*R*)-dihydro-β-ionol acetate (600 MHz, CDCl_3_) ^1^H NMR (600 MHz, CDCl_3_) δ (ppm): 0.97 (6H, d, *J* = 5.0 Hz), 1.25 (3H, d, *J* = 6.5 Hz), 1.39–1.41 (2H, m), 1.53–1.65 (8H, m), 1.91–199 (4H, m), 2.05 (3H, s), 4.88–4.93 (1H, sextet, *J* = 2.0 Hz); ^13^C NMR (151 MHz, CDCl_3_) δ (ppm): 19.5, 19.7, 21.3, 24.2, 28.5, 32.7, 34.9, 36.4, 39.8, 68.8, 127.2, 136.5, 170.8.

### 3.3. Microwave-Assisted Deacetylation of (R)-Dihydro-β-ionol Acetate

A 10 mL microwave reaction vessel was charged with 3.0 mL of 25% sodium hydroxide solution, 3.0 mL of ethanol, and 1.26 mmol of (*R*)-dihydro-β-ionol acetate. The vessel was sealed and heated under microwave-assisted heating in a microwave reactor (Biotage Initiator+) at 80 °C for 30 min with constant magnetic stirring. After the completion of the reaction, the mixture was cooled, and the product was extracted with dichloromethane (5 × 10 mL). The combined organic layers were dried over anhydrous sodium sulfate, and the dichloromethane was removed by evaporation under reduced pressure. The resulting (*R*)-dihydro-β-ionol was obtained, as a colorless oil, with sufficient purity for direct use without further purification, (246.9 mg, >99% e.e, yield 100%).

(*R*)-dihydro-β-ionol ^1^H NMR (600 MHz, CDCl_3_) δ (ppm): 1.00 (6H, s), 1.22 (3H, d, *J* = 6.5 Hz), 1.42–1.44 (2H, m), 1.49–1.61 (8H, m), 1.90 (2H, t, *J* = 6.0 Hz), 1.92–1.98 (1H, td, *J* = 2.8 Hz), 2.11–2.16 (1H, td, *J* = 2.8 Hz), 3.78–3.83 (1H, sextet, *J* = 2.1Hz); ^13^C NMR (151 MHz, CDCl_3_) δ (ppm): 19.5, 19.7, 23.2, 24.7, 28.5, 32.7, 34.9, 39.8, 39.9, 68.8, 126.9, 136.8; [α]^26^_D_-3,5 (c = 0,15, CHCl_3_).

### 3.4. Intramolecular Microwave-Assisted Cyclization for the Synthesis of (2R,5S)-Theaspirane

In a 10 mL glass tube, the corresponding alcohol (**5**) (1.0 mmol, 196.0 mg), selenium dioxide (SeO_2_, 2.0 mmol, 220.0 mg), and 5 mL of dry 2-methyltetrahydrofuran was placed in a microwave reactor (Biotage Initiator+) and magnetically stirred at 80 °C for 30 min. After cooling, the reaction mixture was filtered over a Celite pad to remove black selenium, and the filtrate was extracted with dichloromethane (3 × 10.0 mL). The combined organic layers were concentrated under reduced pressure, and the residue was purified by column chromatography on neutral alumina using a mixture ranging from 100:0 to 90:10 (hexane/ethyl acetate as the eluent. The (2*R*,5*S*)-theaspirane was isolated as colorless oil, (137.9 mg, >99% e.e, >99% d.e yield 71%).

(2*R*,5*S*)-theaspirane (600 MHz, CDCl_3_) ^1^H-NMR δ: 0.87 (3H, s), 0.99 (3H, s), 1.28 (3H, d, *J* = 6.0 Hz), 1.31 (1H, dt, *J* = 5.3, 13.0 Hz), 1.62–1.53 (1H, m), 1.68 (1H, dt, *J* = 15.1, 7.6 Hz), 1.72 (3H, bs), 1.81 (1H, m), 2.12–1.96 (4H, m), 4.03 (1H, sextet, *J* = 2.8 Hz), 5.42 (1H, m); ^13^C NMR (151 MHz, CDCl_3_) δ: 19.4, 21.3, 22.9, 24.0, 31.3, 33.7, 36.2, 37.9, 76.6, 87.6, 123.9, 136.8; [α]^24^_D_-175 (c = 0.20, CHCl_3_).

### 3.5. Intramolecular Microwave-Assisted Cyclization for the Synthesis of Racemic Theaspirane—Scale up

In a 20 mL glass tube, the corresponding alcohol (**2**) (10.0 mmol 1.96 g), selenium dioxide (SeO_2_, 20.0 mmol, 2.20 g), and 15 mL of dry 2-methyltetrahydrofuran was placed in a microwave reactor (Biotage Initiator+) and magnetically stirred at 80 °C for 30 min. After cooling, the reaction mixture was filtered over a Celite pad to remove black selenium, and the filtrate was extracted with dichloromethane (3 × 15.0 mL). The combined organic layers were concentrated under reduced pressure, and the residue was purified by column chromatography on neutral alumina using a mixture ranging from 100:0 to 90:10 (hexane/ethyl acetate as the eluent. The racemic theaspirane was isolated as colorless oil, (1.36 g, yield 70%).

(2*R*,5*S*)-theaspirane (600 MHz, CDCl_3_) ^1^H-NMR δ: 0.87 (3H, s), 0.99 (3H, s), 1.28 (3H, d, *J* = 6.0 Hz), 1.31 (1H, dt, *J* = 5.3, 13.0 Hz), 1.62–1.53 (1H, m), 1.68 (1H, dt, *J* = 15.1, 7.6 Hz), 1.72 (3H, bs), 1.81 (1H, m), 2.12–1.96 (4H, m), 4.03 (1H, sextet, *J* = 2.8 Hz), 5.42 (1H, m); ^13^C NMR (151 MHz, CDCl_3_) δ: 19.4, 21.3, 22.9, 24.0, 31.3, 33.7, 36.2, 37.9, 76.6, 87.6, 123.9, 136.8.

## 4. Conclusions

In conclusion, the optimization of the (2*R*,5*S*)-theaspirane synthesis, a key kairomone for the banana weevil, was successfully achieved through microwave-assisted reactions and green chemistry principles. This enhanced methodology significantly reduced reaction time, improved stereoselectivity, and minimized the use of toxic reagents, making the process more efficient, environmentally sustainable, and scalable. By addressing long-standing synthetic challenges, this approach not only enhances the feasibility of large-scale production but also offers promising applications in pest management.

Nevertheless, despite the considerable reduction in SeO_2_ usage through a well-optimized and rational approach, further refinements remain possible. The implementation of an in situ catalytic selenium oxidation system could further minimize the molar ratio, ultimately eliminating the need for stoichiometric quantities and enhancing the overall sustainability of the process.

Finally, this study reinforces the critical role of sustainable chemistry in organic synthesis and agricultural applications. Future research should focus on field validation and the integration of (2*R*,5*S*)-theaspirane with other semiochemicals to develop more effective and eco-friendly pest control strategies.

## Figures and Tables

**Table 1:** Optimization of the reaction conditions for cyclization with SeO_2_.

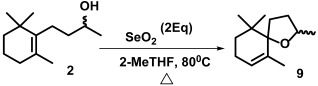					
Entry	Temp. (°C)	Time (min)	Solvent	SeO_2_(Equivalents)	Yield (%) ^b^
1	60 ^a^	90	dioxane	1	12
2	60 ^a^	90	dioxane	2	28
3	60	15	2-MeTHF	1	15
4	60	30	2-MeTHF	1	23
5	60	15	2-MeTHF	2	31
6	60	30	2-MeTHF	2	35
7	80	15	2-MeTHF	1,1	30
8	80	30	2-MeTHF	1,1	33
9	80	15	2-MeTHF	2	54
10	80	30	2-MeTHF	2	73
11	80	60	2-MeTHF	2	40
12	80	30	2-MeTHF	3	56
13	100	15	2-MeTHF	1,1	12
14	100	15	2-MeTHF	2	29
15	100	15	2-MeTHF	4	25
16	100	30	2-MeTHF	2	0

^a^ Conventional heating. ^b^ The yields were measured after the products were isolated and purified by column chromatography.

**Table 2:** Comparative analysis of reaction conditions, yields, enantiomeric excess values, and, when applicable, the enantiomeric ratio (E), for previously published reactions and the new methodology.

Entry	Reagents and Conditions	Overall Yield (%)	e.e (%)	Enantiomeric Ratio (E) ^c^	Reference
1	AlCl_3_.6H_2_O; Na/NH_3_; CuBr_2_, CaCO_3_. ^a^	20	Mixture isomers	-	[11]
2	bis(*p*-chloro phenyl diselenide), Et_4_NCl_4_/electrode Pt. ^a^	70	Mixture isomers	-	[13]
3	LiAlH_4_, CCl_4_, H_2_, Pd/BaSO_4_, acidic conditions, energy-intensive, techniques, prolonged reaction times.	did not report the yields for all products.	98%	-	[16]
4	Ph_3_SnH, CrO_3_, t-BuOOH, benzene, NaBH_4_, CeCl3.7H_2_O, amberlyst 15, −20 °C. ^a^	46	Mixture isomers	-	[17]
5	AIBN, PhSH, MCPBA, propylene oxide, LDA, cryogenic temperatures. ^a^	21	Mixture isomers	-	[19]
6	Dioxane, SeO_2_, (No quantities or methodologies described), prolonged reaction times. ^a^	41 *	98.7 *	1377	[21]
7	2-MeTHF, optimized methodologies for kinetic resolution and cyclization (SeO_2_), decreased total reaction time. ^b^	71	99.9	15,198	This work

* Chromatographic separation presented significant difficulty. ^a^ Reaction under conventional heating. ^b^ Reaction under microwave-assisted conditions. ^c^ Calculated according to Gawley [23].

## Data Availability

Data are contained within the article and Supplementary Materials.

## Supplementary Materials

The following supporting information can be downloaded at: https://www.mdpi.com/article/10.3390/molecules30071519/s1, detailed optical rotation, ^1^H NMR and ^13^C NMR spectral data for compounds **2**–**5**, **7** and **9** Figures S1–S15, Scheme S1, is reported in reference [16,21,24].

## Abbreviations

The following abbreviations are used in this manuscript:

^1^HNMR	proton nuclear magnetic resonance
^13^CNMR	Carbon-13 nuclear magnetic resonance
GC-FID	gas chromatography with flame ionization detection
