# Supplementary material for: Microwave-Assisted Enantioselective Synthesis of (2R,5S)-Theaspirane: A Green Chemistry Approach
Source: Molecules. 2025 Mar 29;30(7):1519. doi: 10.3390/molecules30071519 (PMC11990196; doi:10.3390/molecules30071519)
Supplement: Supplementary file 1 [file molecules-30-01519-s001.zip › molecules-3515527-supplementary.pdf]

Supplementary Material

Microwave-Assisted Enantioselective Synthesis of (2*R*,5*S*)-Theaspirane: A Green Chemistry Approach

Sayuri Cristina Santos Takada [1,*], Maria Carolina Blassioli-Moraes [1], Miguel Borges [1], Raul Alberto Laumann [1], Izabella Vitória Maravalho [2] and Wender Alves Silva [2,*]

Contents

General Information..S2

Analytical Data...S3

Spectra and Chromatograms...S5

All reagents and solvents were purchased from Sigma-Aldrich Merck. The solvents were from commercial sources and when required were treated and dried as according to the literature. Purification of the products was performed by flash chromatographic column, using silica gel 60, 230-400 mesh ASTM Merck. The reactions were monitored by thin-layer chromatography (TLC) on precoated 0.25mm thick plates of Kieselgel 60 F_{254}; visualization was accomplished by UV light (254 nm) or by spraying a solution of 5 % (w/v) vanillin in 100ml 20 % (w/v) aq. sulfuric acid and heating at 200°C for a sufficient duration until blue spots become visible. The microwave reactions were performed on a Biotage Initiator+ microwave reactor using sealed vessels, dynamic program, temperature detection by internal fiber optic probe, simultaneous cooling, and media stirring. Optical rotation was measured with a Perkin Elmer Lambda 950 UV-Vis-NIR. The NMR spectra were recorded at 25 °C on a Bruker Avance 600 spectrometer (600 MHz for ^{1}H and 151 MHz for ^{13}C) with TMS as an internal standard for deuterated chloroform ($CDCl_3$) as solvent and chemical shifts (δ) are expressed in ppm referenced by the residual solvent signal and the coupling constants (*J*) in Hertz (Hz). To indicate the multiplicity of the signals the following abbreviations were used: s (singlet), d (doublet), t (triplet), q (quadruplet), m (multiplet), dd (doublet of doublets), ddd (doublet of doublet of doublets), ddt (doublet of doublet of triplets), dt (doublet of triplets), td (triplet of doublets) and tt (triplet of triplets). The enantiomeric and diastereomeric ratios were determined using GC/FID were recorded a Shimadzu GC-2010 PRO was equipped with Supelco β-DEX™ 110 column (30 m × 0.25 mm x 0.25 μm). The injector temperature was set at 200 °C, operating in Split mode (30:1), with manual injection of a 1 μL sample. The carrier gas was helium (He) at a constant flow rate of 0.69 mL/min. The detector temperature was maintained at 250 °C. The gas flow rates were as follows: H_2 = 40.0 mL/min, synthetic air = 400.0 mL/min, and N_2 = 30.0 mL/min. For characterization were used GC/MS Shimadzu GC-2010 chromatograph, was equipped with a 5%-phenyl-95%-methylsiloxane (HP-5) capillary column (30 mm × 0.32 mm × 0.25 μm) and utilized helium as the carrier gas at a flow rate of 1.0 μm/min. The oven temperature was programmed to increase from 100 °C to 200°C at a heating rate of 3°C/min. Data processing was performed using the GC-MS solution software. The melting point were measured with a capillary on a LOGEN Scientific (LS III Plus) apparatus.

Analytical Data

Dihydro-β-ionol (2)

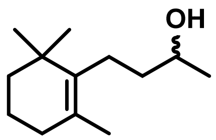

The product was obtained as a white solid, 196.2 mg, yield 100%, m.p 38- 40°C.

^{1}H NMR (600 MHz, $CDCl_3$) δ (ppm): 1.00 (6H, s), 1.22 (3H, d, *J* = 6.5 Hz), 1.42–1.44 (2H, m), 1.49–1.61 (8H, m), 1.90 (2H, t, *J* = 6.0 Hz), 1.92–1.98 (1H, td, *J* = 2.8 Hz), 2.11–2.16 (1H, td, *J* = 2.8 Hz), 3.78–3.83 (1H, sextet, *J* = 2.1Hz); ^{13}C NMR (151 MHz, $CDCl_3$) δ (ppm): 19.5, 19.7, 23.2, 24.7, 28.5, 32.7, 34.9, 39.8, 39.9, 68.8, 126.9, 136.8.

(*S*)-dihydro-β-ionol (3)

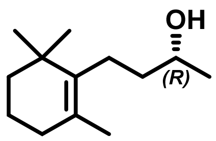

The product was obtained as a colorless oil 100% yield, 246.9 mg.

^{1}H NMR (600 MHz, $CDCl_3$) δ (ppm): 1.00 (6H, s), 1.22 (3H, d, *J* = 6.5 Hz), 1.42–1.44 (2H, m), 1.49–1.61 (8H, m), 1.90 (2H, t, *J* = 6.0 Hz), 1.92–1.98 (1H, td, *J* = 2.8 Hz), 2.11–2.16 (1H, td, *J* = 2.8 Hz), 3.78–3.83 (1H, sextet, *J* = 2.1Hz); ^{13}C NMR (151 MHz, $CDCl_3$) δ (ppm): 19.5, 19.7, 23.2, 24.7, 28.5, 32.7, 34.9, 39.8, 39.9, 68.8, 126.9, 136.8. $[\alpha]^{26}_{D}$ -3,5 (c=0,15, $CHCl_3$).

(*R*)-dihydro-β-ionol (5)

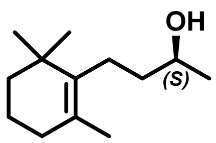

The product was obtained as a colorless oil 48% yield (after kinetic resolution), 14.3 mg.

Same data from compound **2**

(*R*)-dihydro-*β*-ionol acetate (4)

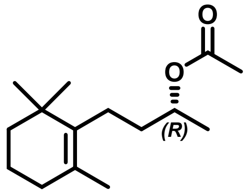

The product was obtained as a colorless oil 49% yield (after kinetic resolution), 17.9 mg.

(600 MHz, $CDCl_3$) ^{1}H NMR (600 MHz, $CDCl_3$) δ (ppm): 0.97 (6H, d, *J* = 5.0 Hz), 1.25 (3H, d, *J* = 6.5 Hz), 1.39–1.41 (2H, m), 1.53–1.65 (8H, m), 1.91-199 (4H, m), 2.05 (3H, s), 4.88-4.93 (1H, sextet,, *J* = 2.0 Hz); ^{13}C NMR (151 MHz, $CDCl_3$) δ (ppm): 19.5, 19.7, 21.3, 24.2, 28.5, 32.7, 34.9, 36.4, 39.8, 68.8, 127.2, 136.5, 170.8.

(2*R*/*S*,5*R*/*S*)-theaspirane (9)

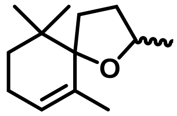

The product was obtained as a colorless oil 73% yield, 141.8 mg.

(600 MHz, $CDCl_3$) ^{1}H-NMR δ: 0.85-0.87 (6H, m), 0.94-0.97 (6H, m), 1.25-1.40 (13H, m), 1.52 – 1.59 (2H, m), 1.76-1.79 (6H, m), 1.80-1.95 (2H, m), 1.97-2.09 (10H, m), 4.00-4.03 (1H, m), 4.11-4.13 (1H, m), 5.25-5.26 (1H, m), 5.40-5.41 (1H, m); ^{13}C NMR (151 MHz, $CDCl_3$) δ: 18.5, 19.4, 21.3, 21.9 22.8, 23.0, 24.0, 24.7, 31.3, 33.7, 34.3, 35.2, 36.2, 36.6, 37.9, 76.6, 76.8, 87.6, 121.5, 123.9, 136.8, 140.2

(2*R*,5*S*)-theaspirane 7

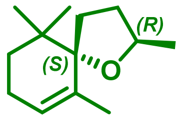

The product was obtained as a colorless oil 71% yield, 137.9 mg.

(600 MHz, $CDCl_3$) ^{1}H-NMR δ: 0.87 (3H, s), 0.99 (3H, s), 1.28 (3H, d, *J* = 6.0 Hz), 1.31 (1H, dt, *J* = 5.3, 13.0 Hz), 1.62 – 1.53 (1H, m), 1.68 (1H, dt, *J* = 15.1, 7.6 Hz), 1.72 (3H, bs), 1.81 (1H, m), 2.12 – 1.96 (4H, m), 4.03 (1H, sextet, *J* = 2.8 Hz), 5.42 (1H, m); ^{13}C NMR (151 MHz, $CDCl_3$) δ: 19.4, 21.3, 22.9, 24.0, 31.3, 33.7, 36.2, 37.9, 76.6, 87.6, 123.9, 136.8; $[\alpha]^{24}_D$ -175 (c=0,20, $CHCl_3$).

Spectra and Chromatograms

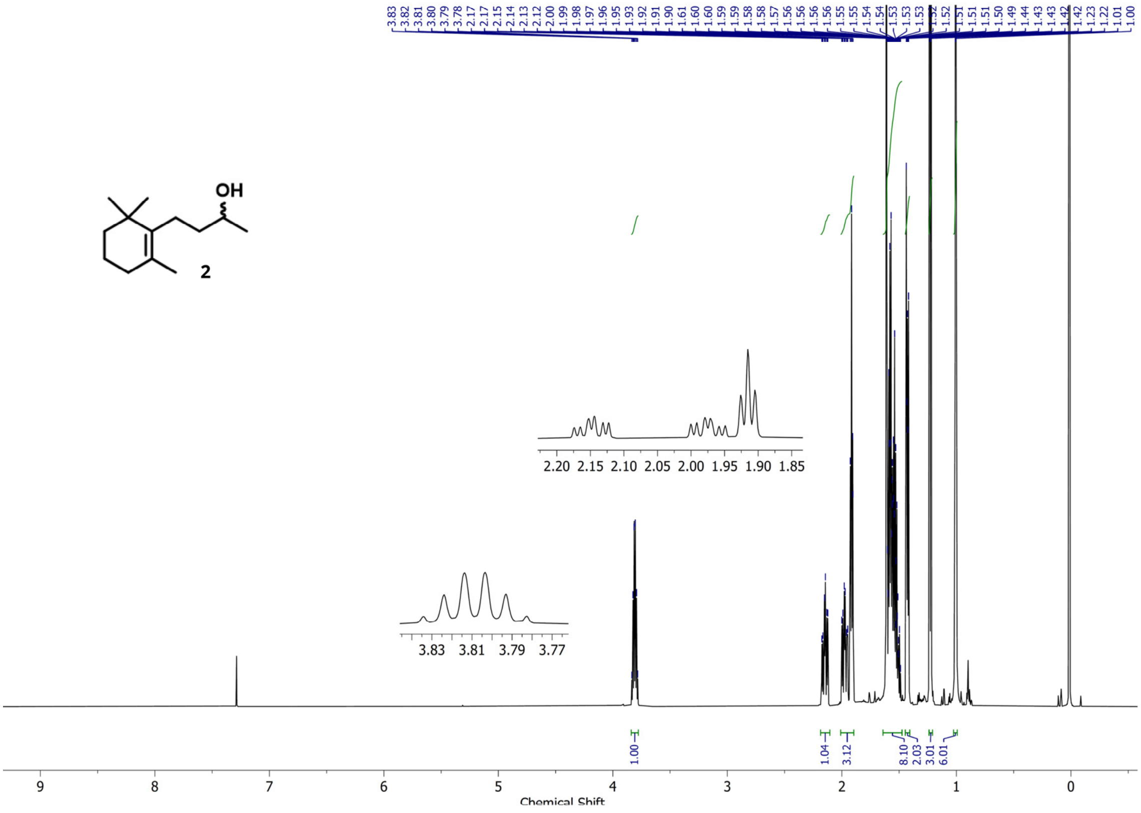

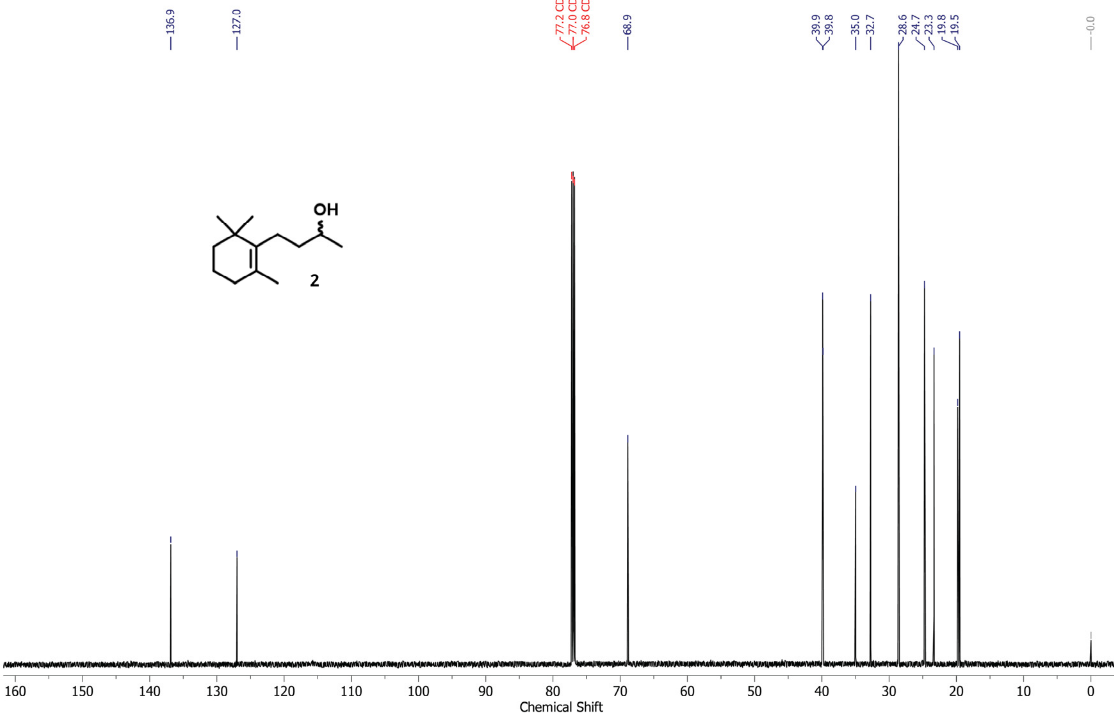

Figure S1. ^{1}H NMR (600 MHz, $CDCl_3$) and ^{13}C NMR (151 MHz, $CDCl_3$) of **2**.

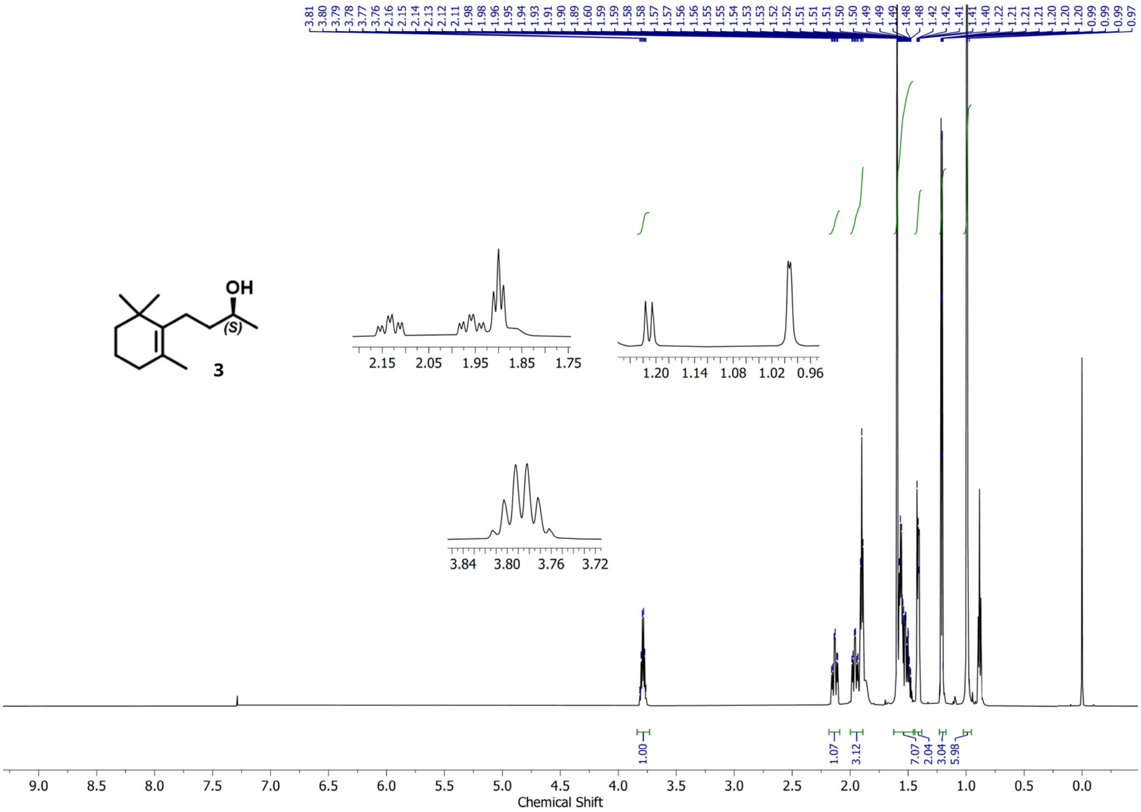

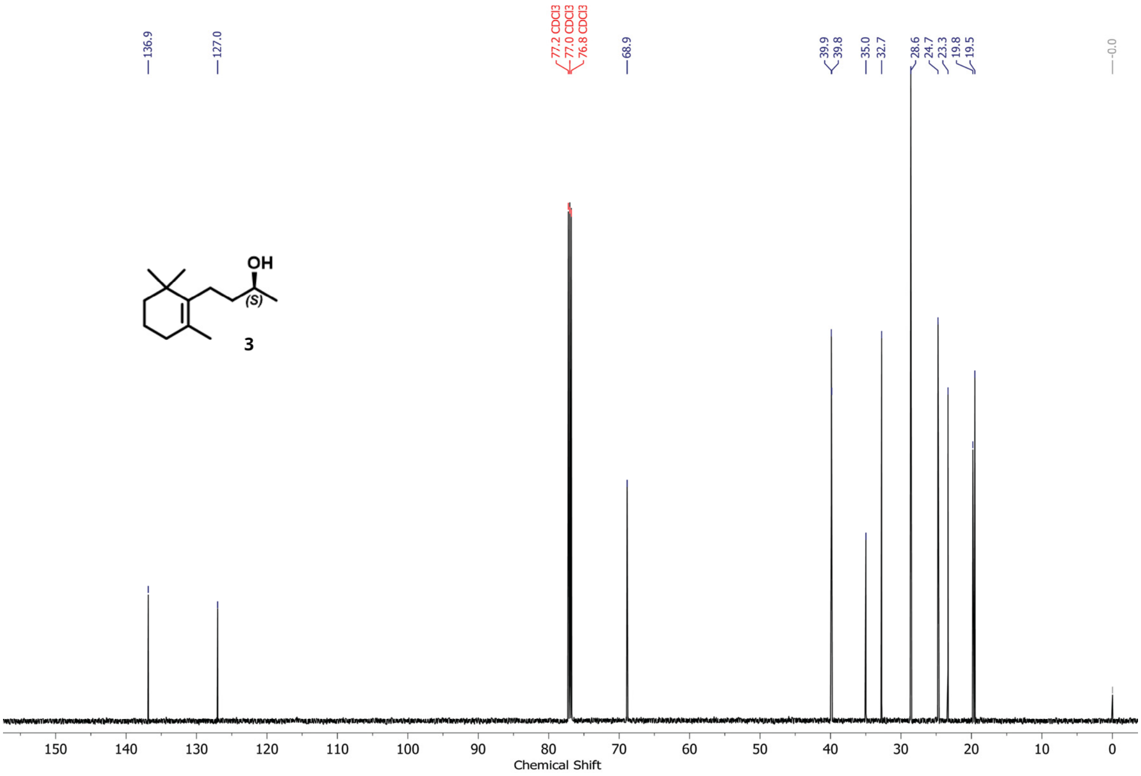

Figure S2. ^{1}H NMR (600 MHz, $CDCl_3$) and ^{13}C NMR (151 MHz, $CDCl_3$) of **3**.

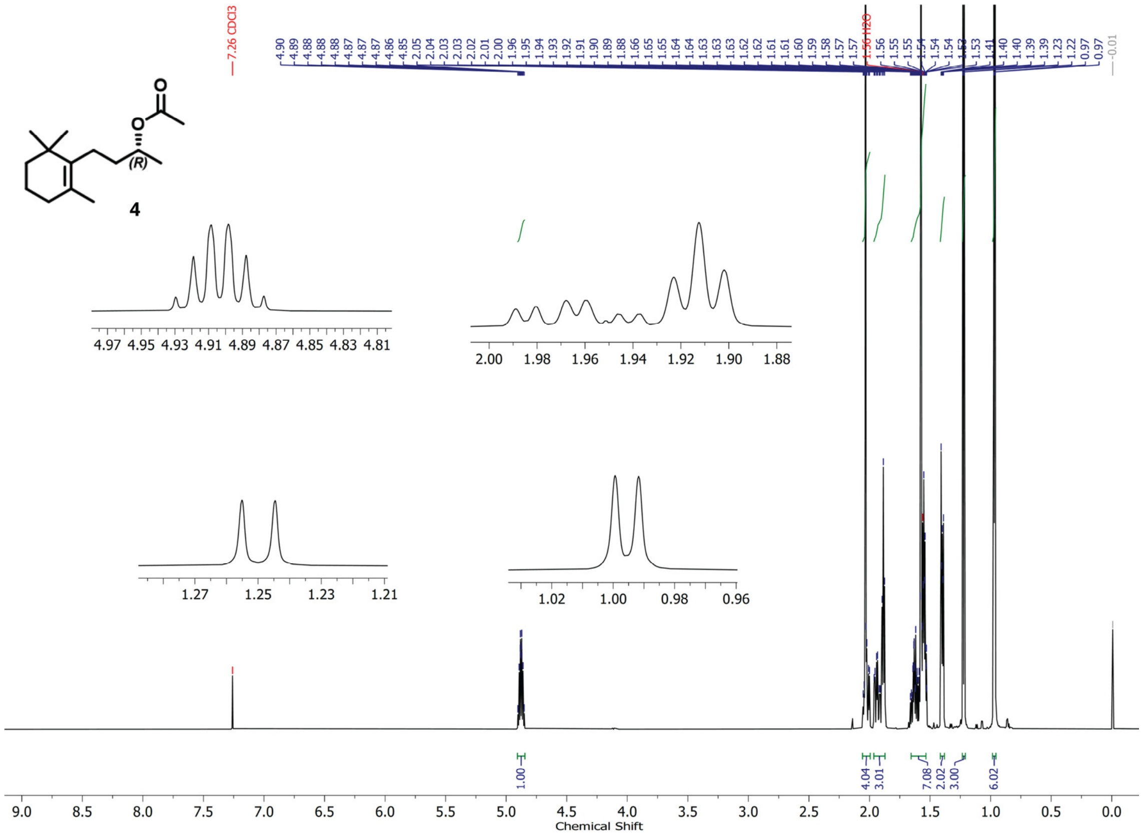

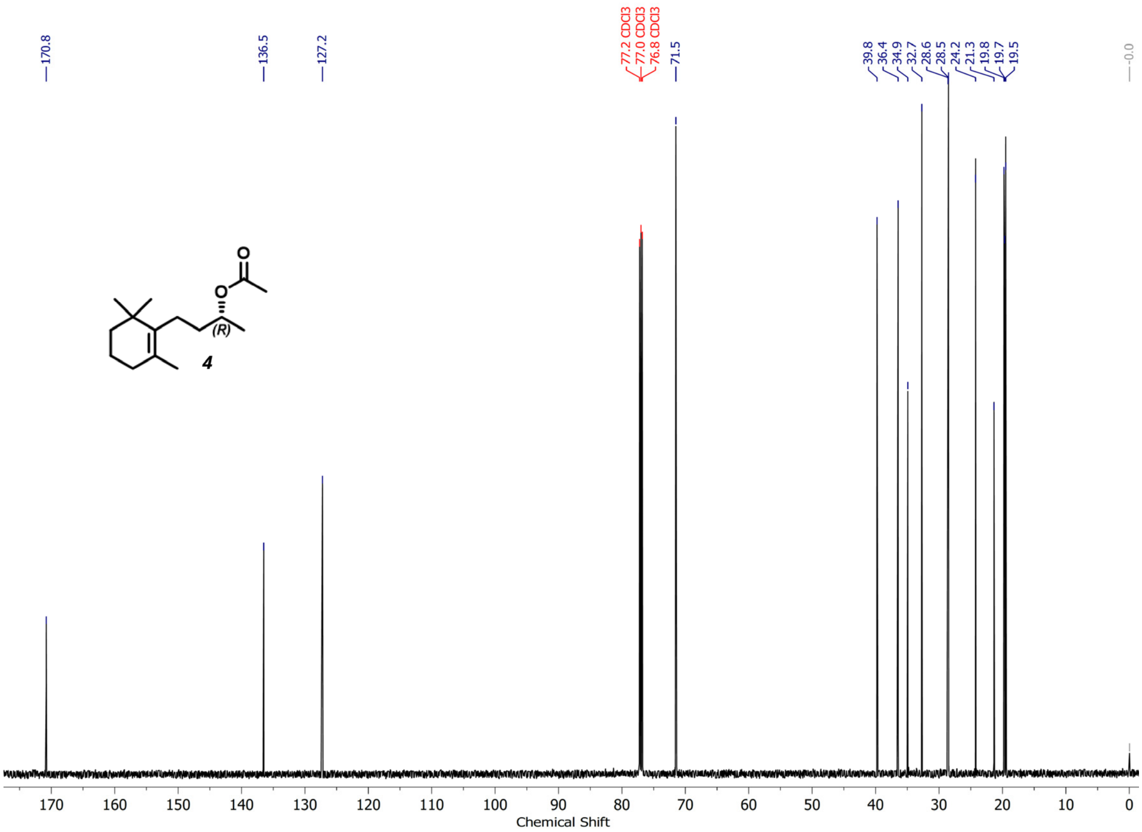

Figure S3. ^{1}H NMR (600 MHz, $CDCl_3$) and ^{13}C NMR (151 MHz, $CDCl_3$) of **4**.

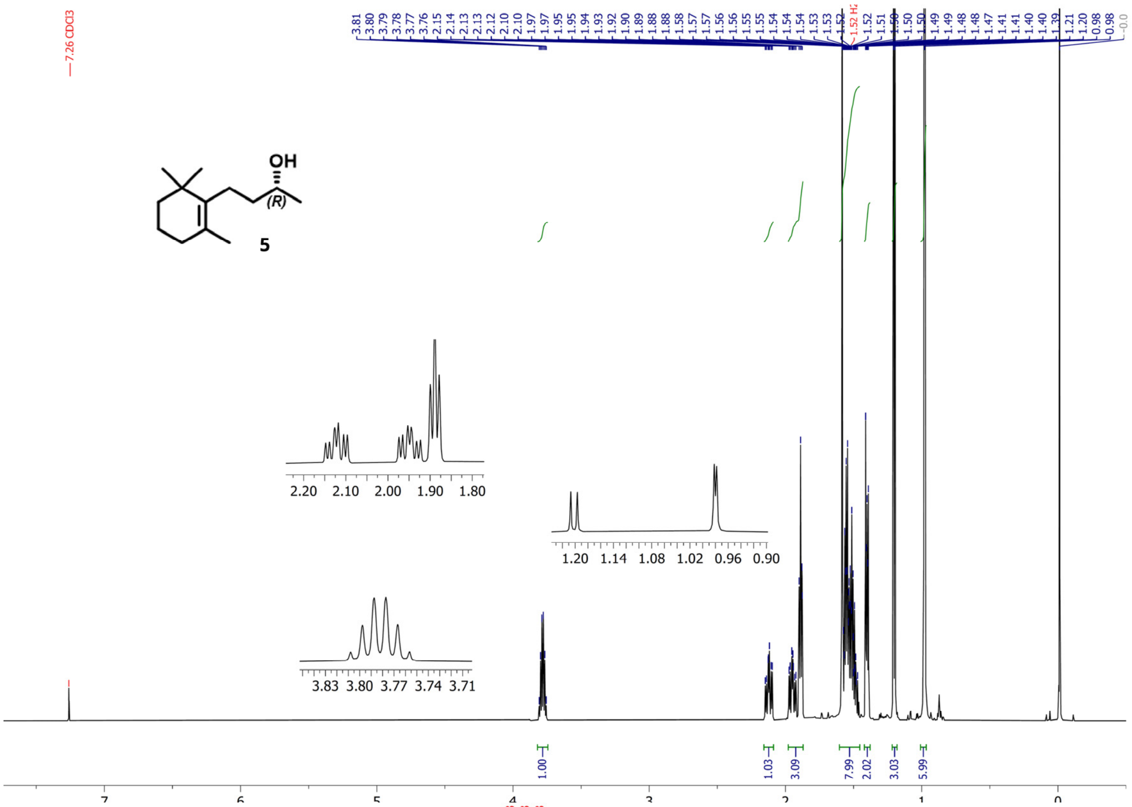

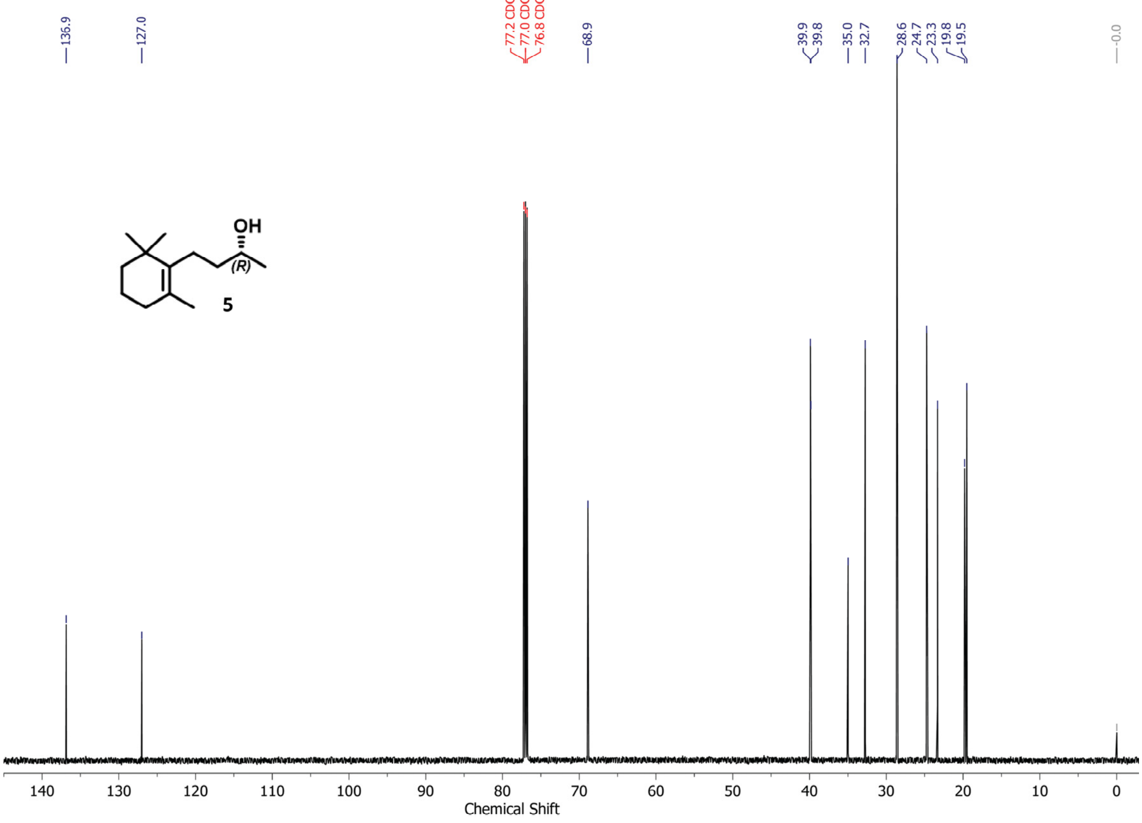

Figure S4. ^{1}H NMR (600 MHz, $CDCl_3$) and ^{13}C NMR (151 MHz, $CDCl_3$) of **5**.

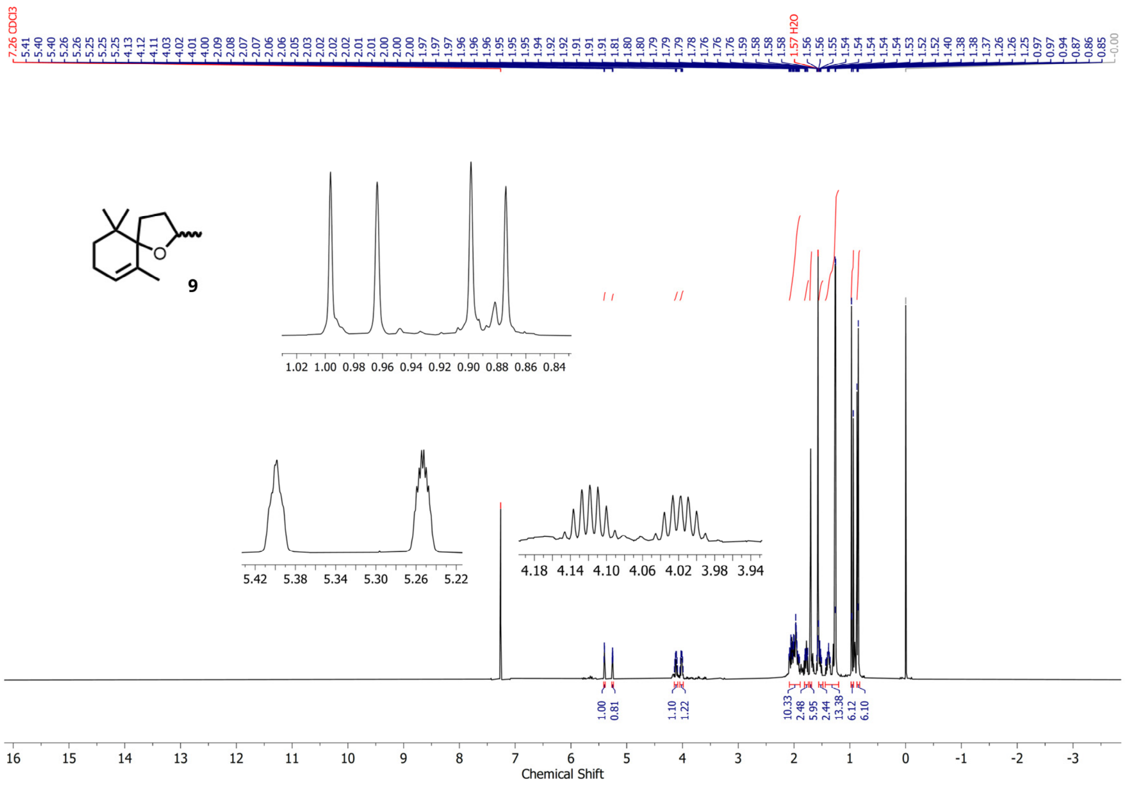

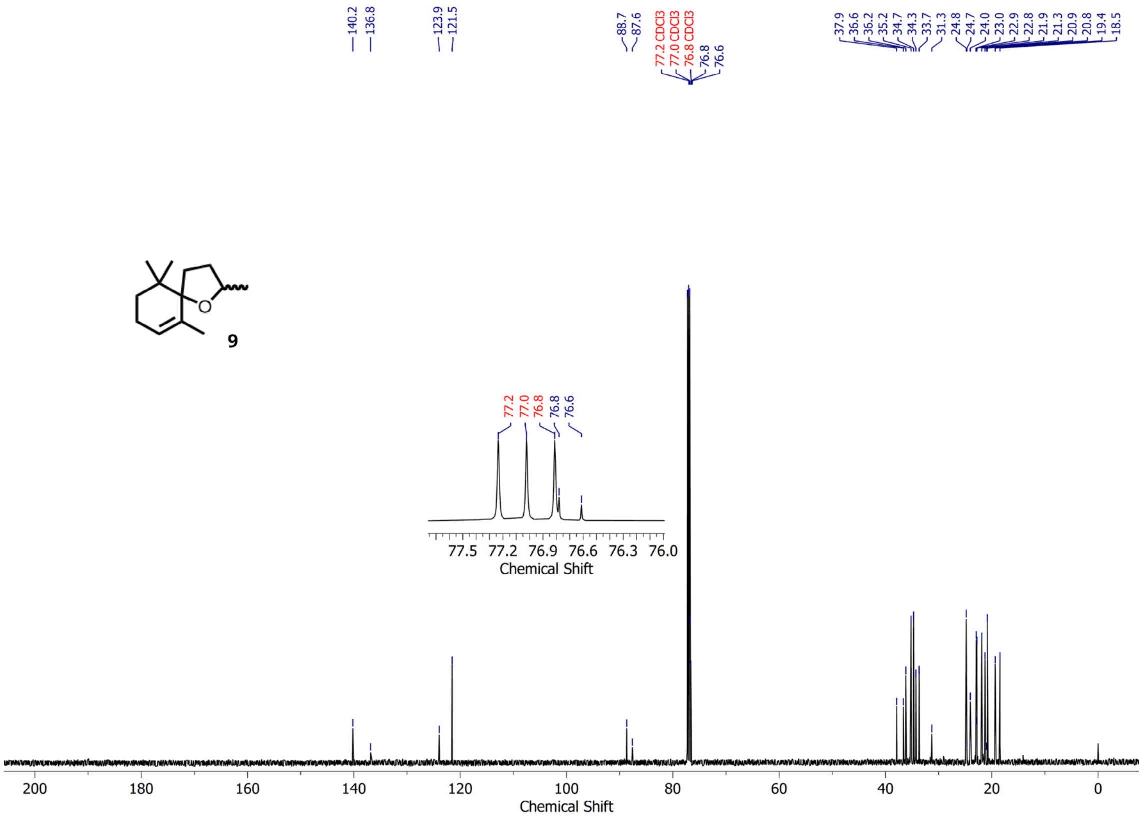

Figure S5. ^{1}H NMR (600 MHz, $CDCl_3$) and ^{13}C NMR (151 MHz, $CDCl_3$) of **9**.

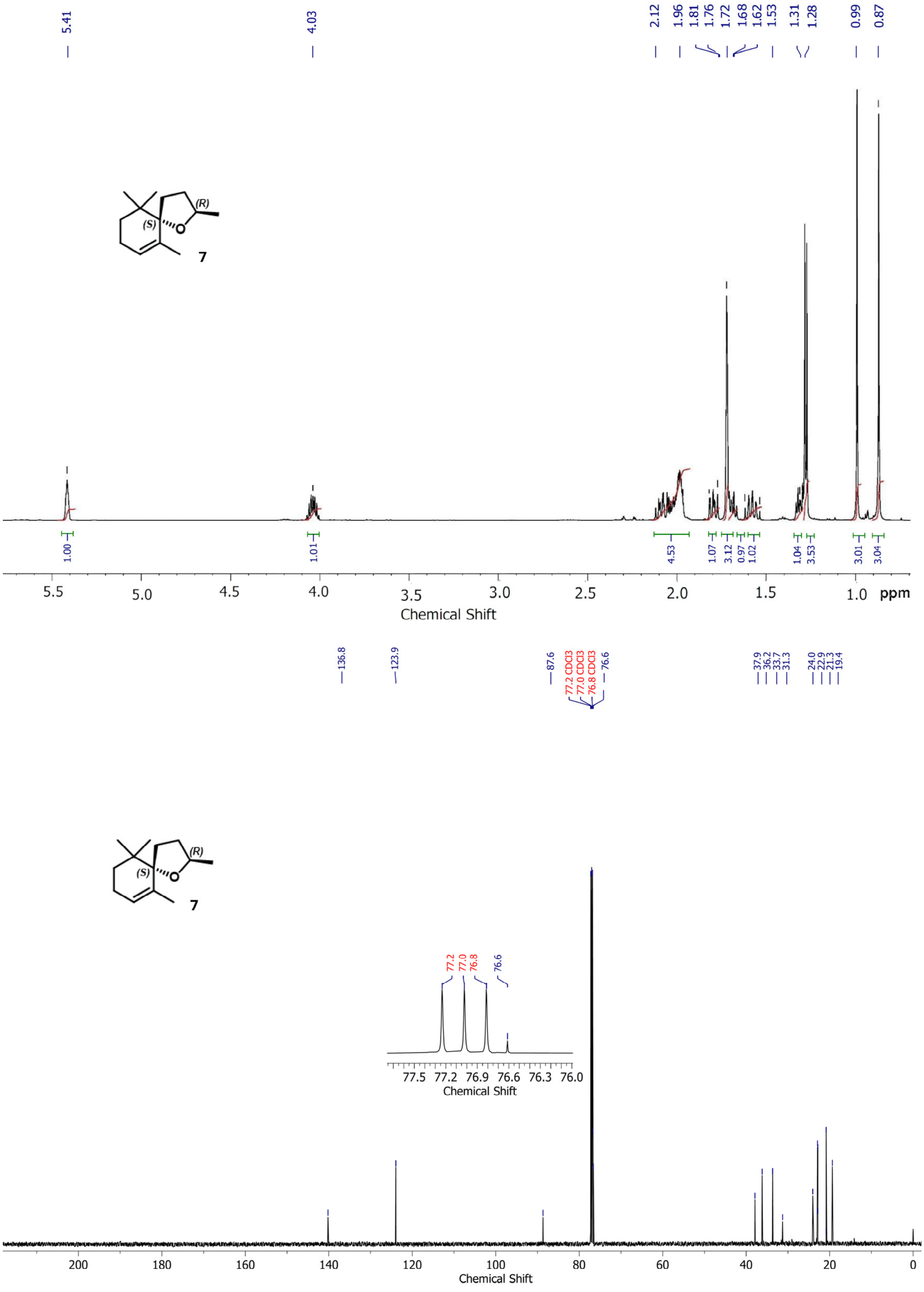

Figure S6. ^{1}H NMR (600 MHz, $CDCl_3$) and ^{13}C NMR (151 MHz, $CDCl_3$) of **7**.

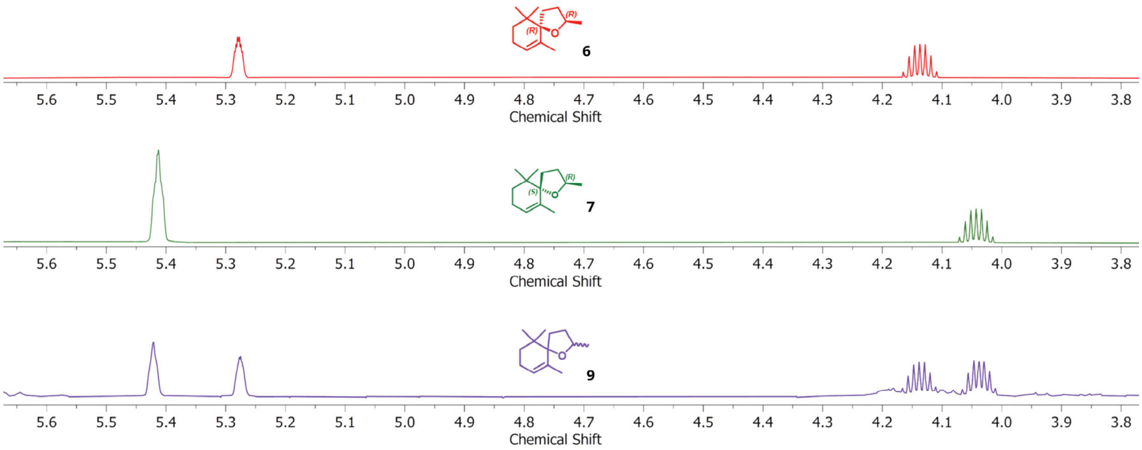

Figure S7. Comparison of the ^{1}H NMR spectra (600 MHz, $CDCl_3$) of compounds a) **6**, (upper) b) **7** (center) and c) **9** (bottom) – characteristic signals of the chiral centers.

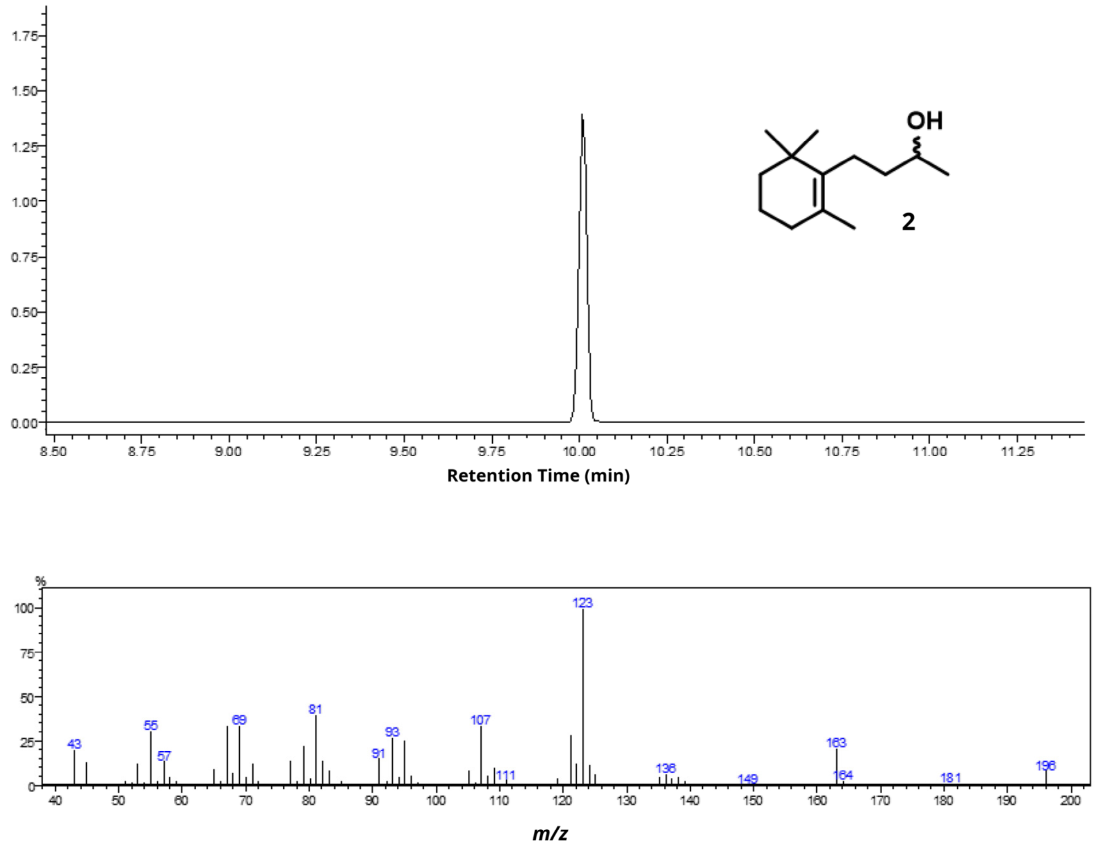

Figure S8. Gas chromatography-mass spectroscopy (GC-MS) chromatograms of compound **2**.

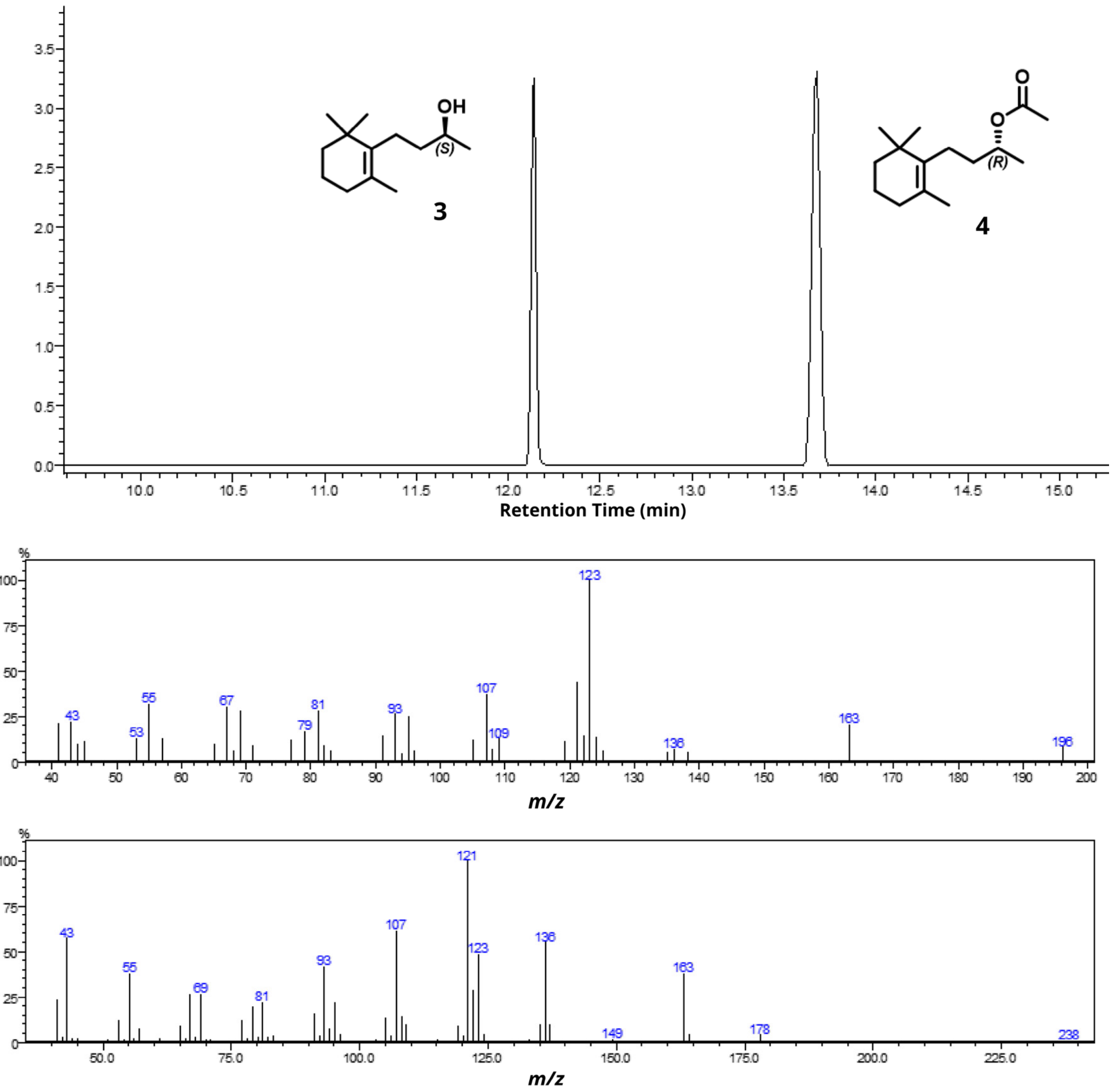

Figure S9. Gas chromatography-mass spectroscopy (GC-MS) chromatograms of compounds **3** and **4** respectively.

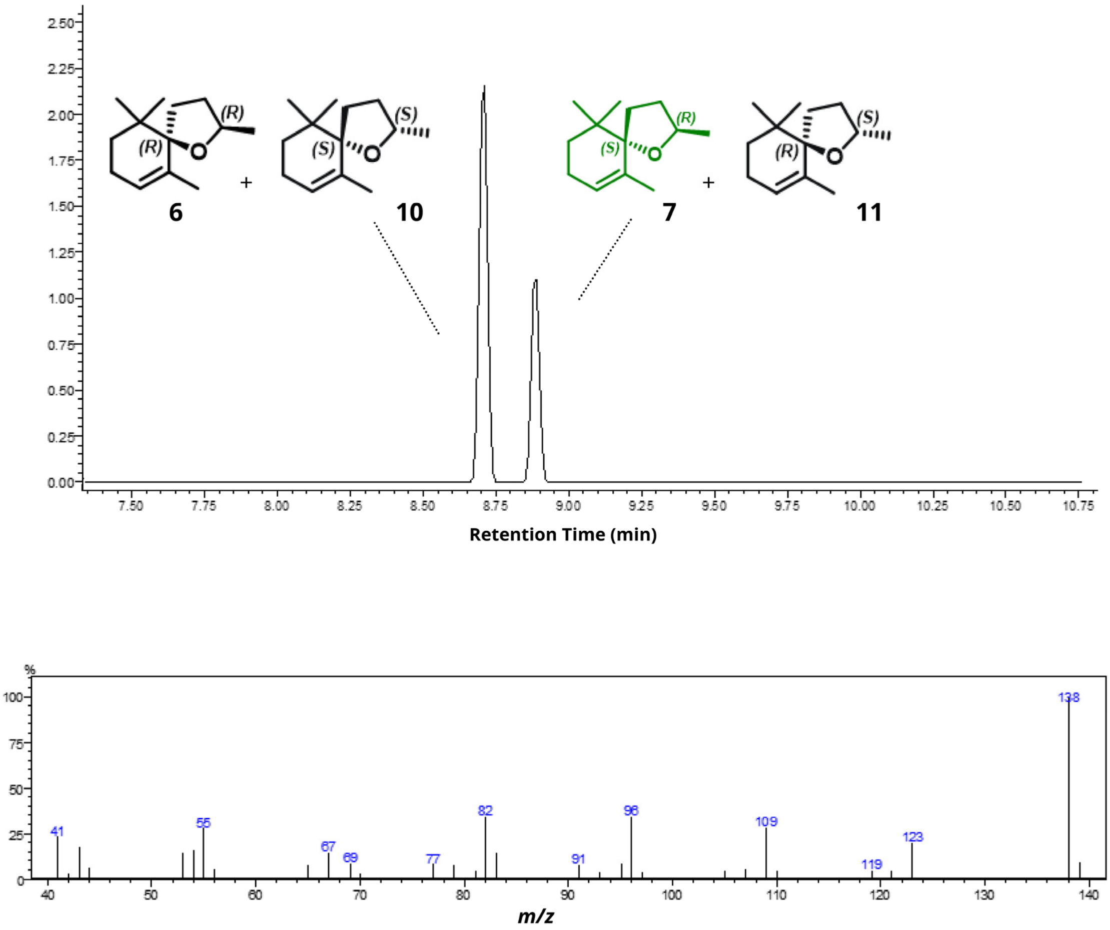

Figure S10. Gas chromatography-mass spectroscopy (GC-MS) chromatograms of compounds **6**, **7**, **8** and **9**.

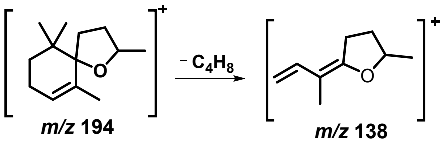

Scheme S1. The ion of *m/z* 138 value results from the retro-Diels–Alder rearrangement of the theaspirane molecular ion of *m/z* 194.

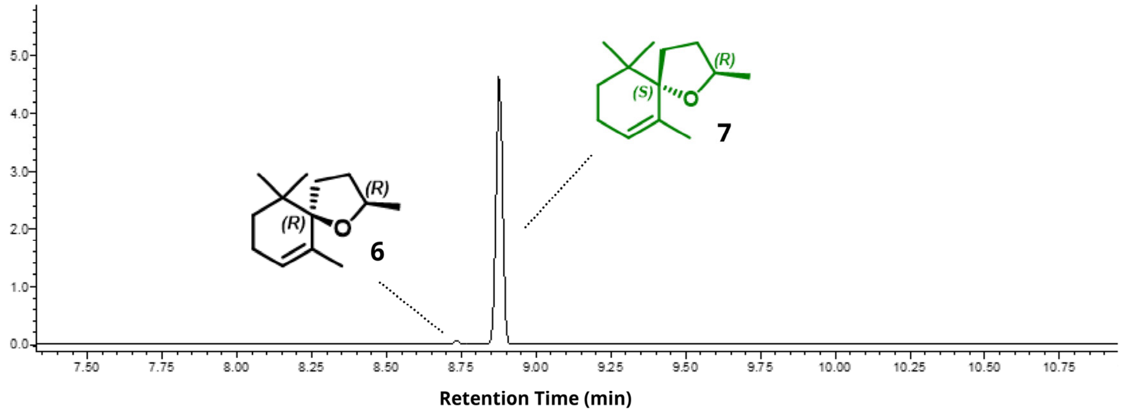

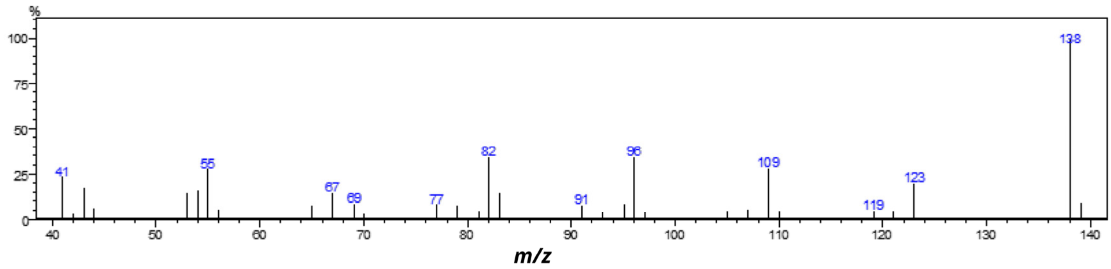

The ion of *m*/*z* 138 value results from the retro-Diels–Alder rearrangement of the theaspirane molecular ion of *m*/*z* 194.

Figure S11. Gas chromatography-mass spectroscopy (GC-MS) chromatograms of compounds **6** and **7**.

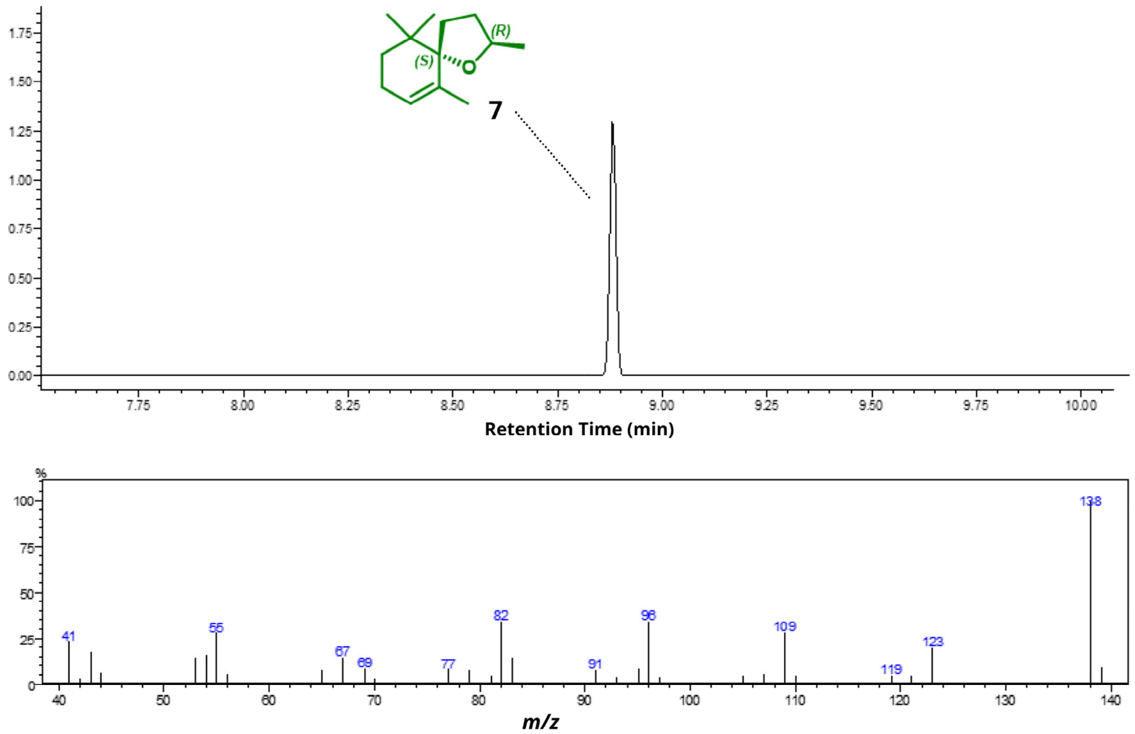

The ion of *m*/*z* 138 value results from the retro-Diels–Alder rearrangement of the theaspirane molecular ion of *m*/*z* 194.

Figure S12. Gas chromatography-mass spectroscopy (GC-MS) chromatograms of compounds **7**.

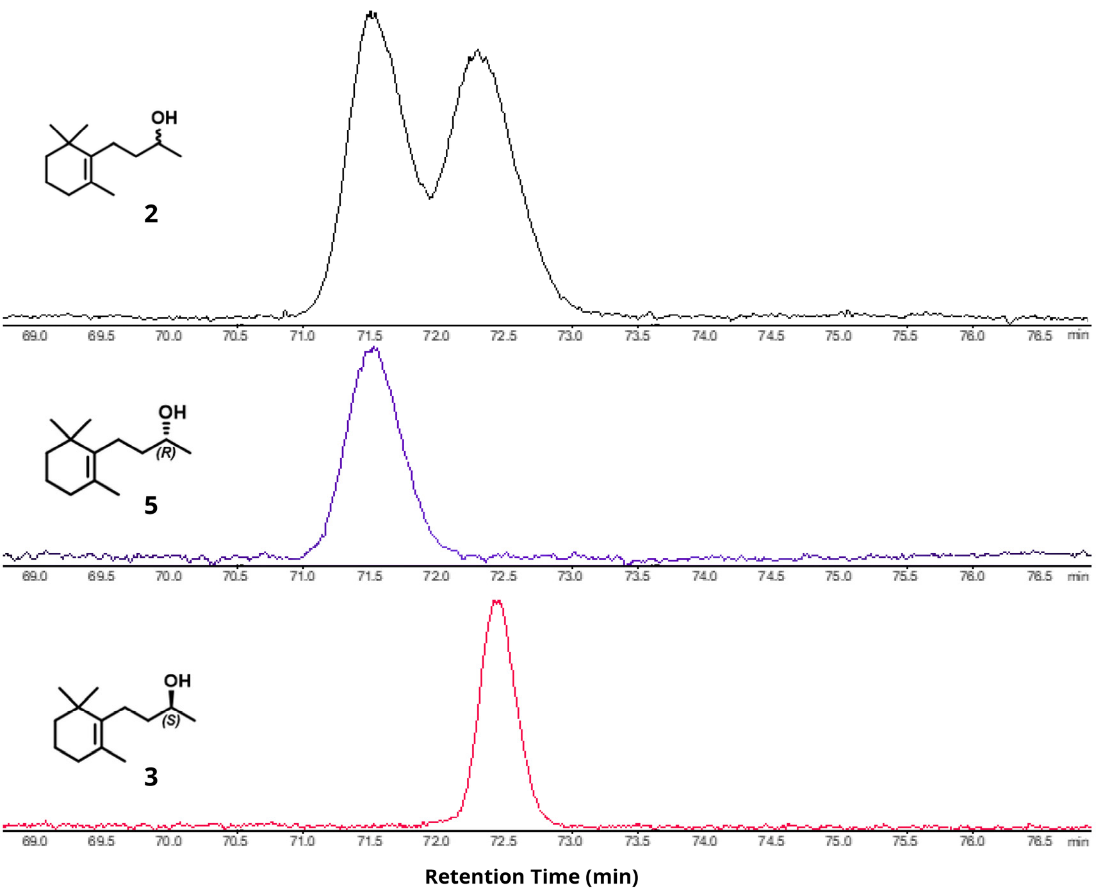

Figure S13. Gas chromatography (GC-FID) chromatograms a) compounds **2** (upper); b) compounds 5 (center); c) compound **3** (bottom).

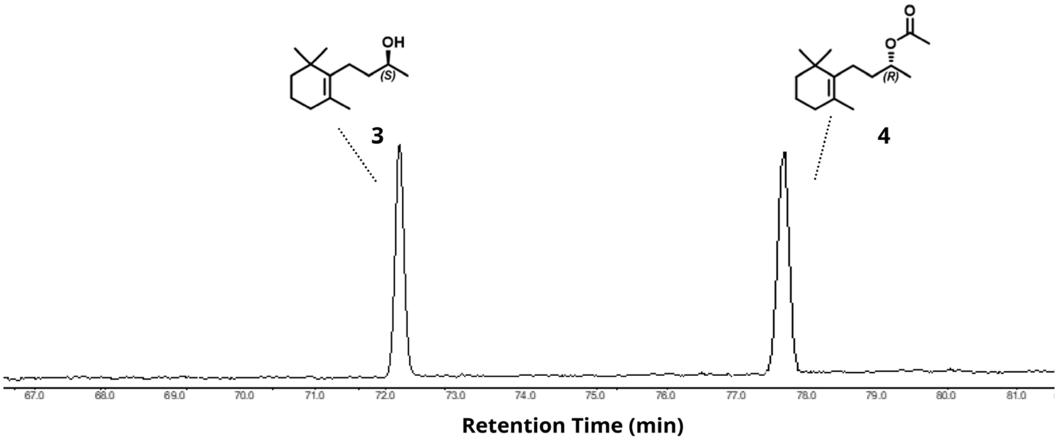

Figure S14. Gas chromatography (GC-FID) chromatograms of compounds **3** and **4.**

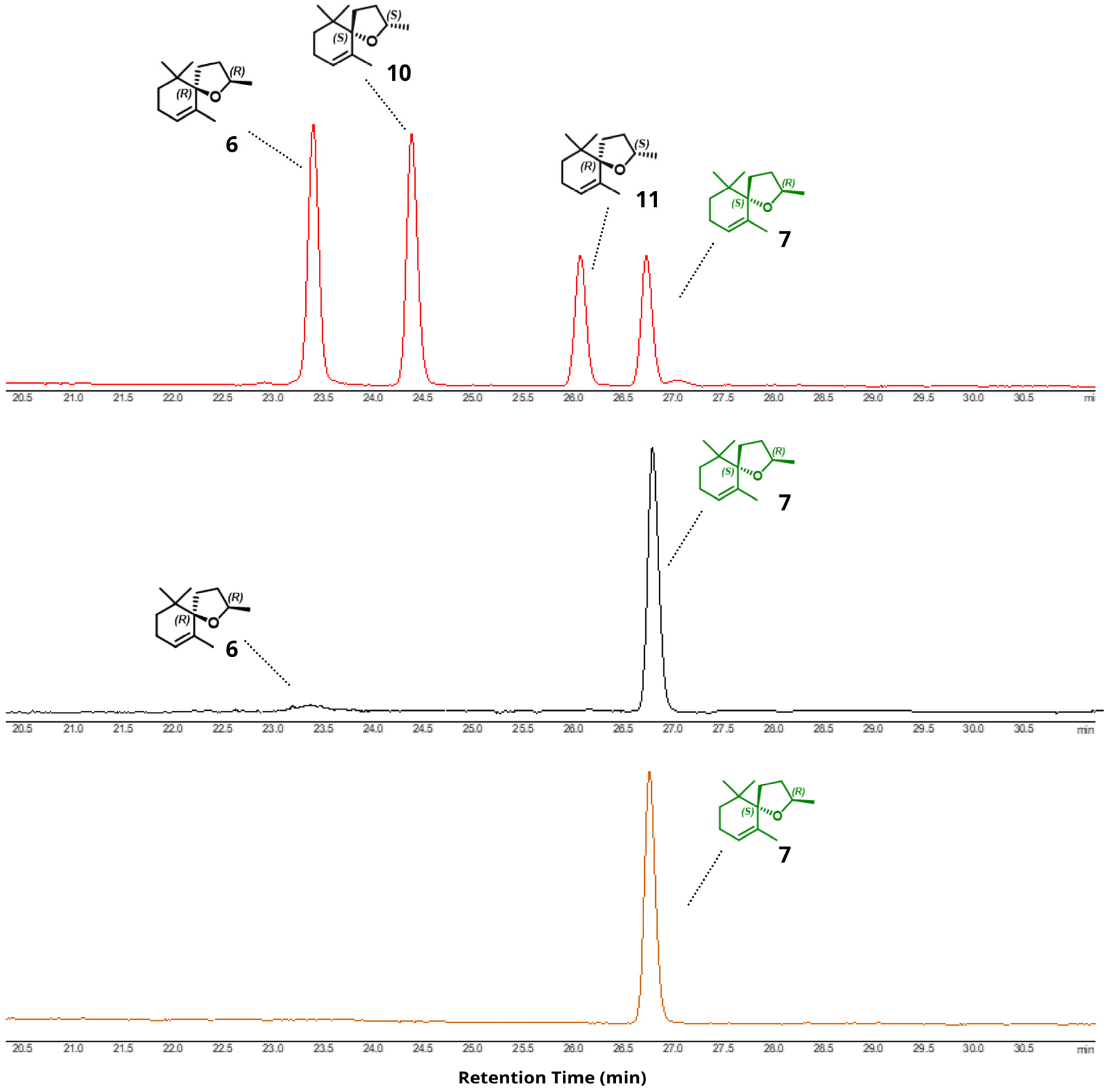

Figure S15. Gas chromatography (GC-FID) chromatograms a) compounds **6**, **7**, **8** and **9** (upper); b) compounds **6** and **7** (center); c) compound **7** (bottom).
